# Versatile electrification of two-dimensional nanomaterials in water

**DOI:** 10.1038/s41467-019-09708-7

**Published:** 2019-04-10

**Authors:** Benoît Grosjean, Marie-Laure Bocquet, Rodolphe Vuilleumier

**Affiliations:** PASTEUR, Département de chimie, École normale supérieure, PSL University, Sorbonne Université, CNRS, 24 Rue Lhomond, 75005 Paris, France

## Abstract

The recent emergence of nanofluidics has highlighted the exceptional properties of graphene and its boron-nitride counterpart as confining nanomaterials for water and ion transport. Surprisingly, ionic transport experiments have unveiled a consequent electrification of the water/carbon surfaces, with a contrasting response for its water/boron-nitride homologue. In this paper, we report free energy calculations based on ab initio molecular dynamics simulations of hydroxide OH^−^ ions in water near graphene and hexagonal boron nitride (h-BN) layers. Our results disclose that both surfaces get charged through hydroxide adsorption, but two strongly different mechanisms are evidenced. The hydroxide species shows weak physisorption on the graphene surface while it exhibits also strong chemisorption on the h-BN surface. Interestingly OH^−^ is shown to keep very fast lateral dynamics and interfacial mobility within the physisorbed layer on graphene. Taking into account the large ionic surface conductivity, an analytic transport model allows to reproduce quantitatively the experimental data.

## Abstract (web summary)

The origins of the different charging processes observed in graphene and boron-nitride nanofluidics are still under debate. Here, using ab-initio molecular dynamics, the authors show that hydroxide species in water exhibits physisorption on graphene but strong chemisorption on boron-nitride.

## Introduction

The common wisdom states the chemical inertness of boron-nitride and carbon-based nanomaterials in mild conditions^1,2^. However, this passive picture is not a dogma and was actually invalidated in various conditions. For example, defect-free graphene basal plane can be activated electronically when supported on metal substrates in ultrahigh vacuum^3^. More puzzling, the chemical reactivity of carbon and boron-nitride two-dimensional materials was assessed in aqueous solvents, thanks to nanofluidic transport measurements. Indeed giant surface charging was highlighted in ionic transport measurement of electrolytes through multiwall nanotubes made from carbon (CNT) or its BN analogue (BNNT)^4,5^, with a surface charge reaching up to 1 C.m^−2^ for BNNT and 0.01 to 0.1 C.m^−2^ for CNT. This is one to two orders of magnitude larger than any other reported surface charge, e.g., on silica glass^6^. The strong pH dependence for both materials led experimentalists to suggest hydroxide adsorption as a source of surface charge for both materials, although the very different salinity dependence pointed to different mechanisms and strengths of adsorption. This conjecture remains however unsupported up to now in terms of the chemical reactivity of these materials and the fundamental origin of this surface charging remains mysterious. Previous theoretical explorations of the behaviour of hydroxide ions at hydrated graphenic surfaces were developed at the semi-empirical level using classical dynamics (MD) study^7^. However, this level of modelling cannot correctly describe OH^−^ in a hydrogen-bonded water network as it is eager to capture a proton from neighbouring water molecules via the Grotthus mechanism. Recently, a static quantum chemistry calculation without explicit water showed that BN can form a stable chemical bond with the hydroxide ion while carbon cannot, in contrast with the experiments and thus deepening the mystery of carbon charging^8^. One key challenge in this quest is, therefore, to properly simulate interfacial OH^−^ ions in water, whose identity remains elusive due to the Grotthus mechanism: this requires an electronic description of the charged hydroxide species, as well as the surrounding water molecules and the reacting surface. Only computationally expensive ab initio molecular dynamic (AIMD), where the nuclei are classically propagated according to forces obtained from an ab initio electronic structure theory, are able to comprehensively reproduce the subtleties of the chemistry of OH^−^ at interfaces. This should build on the numerous AIMD studies of the intrinsic reactivity of hydroxide in bulk water^9–19^, which made considerable progress lately. Despite a dependence on the type of density functional theory (DFT) functional, the current consensus is that of OH^−^ generally forming two hydration states in bulk water with three or four hydrogen bonds donated to the hydroxide oxygen respectively (the latter state being called hypercoordinated). Proton transfer is facilitated when OH^−^ is receiving three hydrogen bonds, but hindered with four bonds. However, simulating accurately the free energy of adsorption of hydrated hydroxide at interfaces is challenging and has been limited to date to the water/air interface^16,20,21^. Also, for this interface, comparison with experiments is delicate because of impurity effects^22^.

Herein we report AIMD-based calculations of the free energy of a hydroxide at the water-graphene and the water-BN interfaces, considering that the clear experimental pH dependency of the surface charge infers a charging due to the anionic self-ion of water rather than its hydronium counterpart. Using advanced biasing methodologies to compute the potential of mean force (PMF) of the OH^−^, we found free energy minima corresponding to physisorption on both materials and chemisorption solely on the h-BN surface. Additional unbiased simulations confirm both the physisorption and chemisorption features of the PMFs and give insights into the solvating picture of the adsorbed hydroxide. Our results unveil fast migration of physisorbed OH^−^ in the lateral direction parallel to the graphene sheet. Hence, we propose a non-classical view of graphene charging through physisorption of mobile hydroxides and derive an analytic model that highlights quantitative agreement with nanofluidic experimental data when the mobility of the surface hydroxide is taken into account.

## Results

### Potential of mean force protocol and results

The difficulty to compute a PMF as a function of hydroxide-surface distance using restrained sampling methods lies in the reactive nature of OH^−^. As illustrated in Supplementary Movies 1 and 2, frequent proton transfers from a solvating water molecule result in a discontinuous motion of the hydroxide species during MD and subsequent loss of information regarding the identity of the hydroxide oxygen O^*^. In order to keep track of the anion, one needs to apply two simultaneous constraints on O^*^ during the simulations: its spatial location with respect to a surface atom and its hydrogen coordination value.

**Fig. 1:**
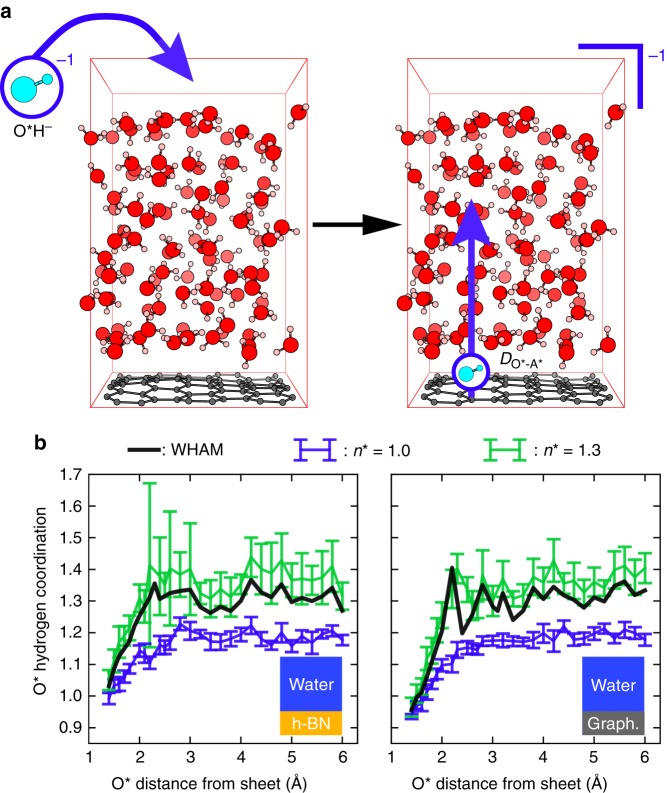
Simulation cell and protocol. **a** Simulation cell: an anionic hydroxide is placed in a 12.83 Å × 12.35 Å × 21.0 Å orthorhombic cell containing a 60 atoms hexagonal graphene sheet in contact with a hundred water molecules (left) resulting in a charged cell (right) in which the hydroxide is progressively displaced (blue vertical arrow) from the surface to the bulk. The hydroxide, hydrogen, carbon and oxygen atoms are respectively represented in cyan, pink, grey and red. **b** Free energy or potential of mean force (PMF) simulation strategy for the water/h-BN and water/graphene interfaces in the constraint’s landscape. For each fixed distance of the hydroxide oxygen O^*^ from the sheets, two MD runs are performed while restraining the hydrogen coordination of O^*^ by a harmonic potential with a target of respectively n^*^ = 1.0 (blue curve) and n^*^ = 1.3 (green curve). The two sets of MD simulations thus obtained are represented by the mean hydrogen coordination and fluctuations around the mean (standard deviations as error bars). They are later combined into a third trajectory labelled WHAM (black curve) using the Weighted Histograms Analysis Method (WHAM) at each ion-surface distance independently

Figure 1a displays the simulation box where one hydroxide with no counterion is inserted into a water/graphene interface (water/h-BN interface not shown) equivalent to an anion concentration of 0.57 M. We propose an original PMF protocol as compared to others^16,23^ schematized in Fig. 1b that we summarize hereafter. The procedure is detailed in the Methods section and in Supplementary Methods. The anion-surface distance $D_{{\mathrm{O}}^ \ast - {\mathrm{A}}^ \ast }$ is fixed by a constraint and is progressively varied by fixed values starting from the surface until the hydroxide has reached the bulk. The hydrogen coordination of the anion oxygen $n_{{\mathrm{O}}^ \ast - {\mathrm{H}}}$ is determined using a switch function (see Supplementary Fig. 1) and is restrained around a target value n^*^ by applying a harmonic potential. Because of the hydroxide solvation varying between the surface and bulk water, its mean coordination takes up values respectively corresponding to 1.0 and 1.3 (see Supplementary Figs. 2 and 3). Two sets of MD trajectories were therefore computed with n^*^ = 1.0 and n^*^ = 1.3, respectively represented by blue and green plots in Fig. 1b. In order to obtain the effective coordination per distance to the surface and accordingly deduce the free energy profile, we apply for each surface-anion distance a standard debiasing method based on the weighted histograms analysis method (WHAM)^24,25^ to the two sets of constrained MD runs. This yields an intertwined set of trajectories labelled WHAM (black lines in Fig. 1b) along which thermodynamic integration is performed to obtain the free energy profile.

**Fig. 2:**
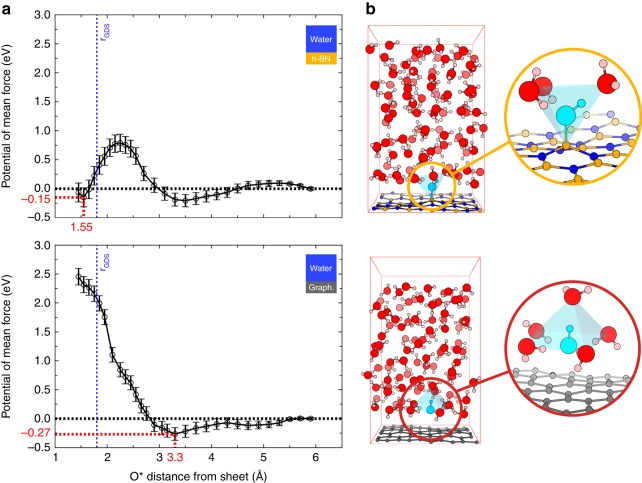
Free energy profiles and stable configurations. **a** Potentials of mean force of an aqueous hydroxide (O^*^H)^−^ with respect to the O^*^ distance from the h-BN (top) and the graphene (bottom) sheets. The position of the Gibbs dividing surface (GDS), derived by adapting the calculation of ref. ^48^, and the position of the free energy reference are respectively indicated by vertical blue and horizontal black dotted lines. Error bars correspond to standard deviations (see Supplementary Methods). **b** Atomistic configurations corresponding to the chemisorbed hydroxide on h-BN (top) and the anion physisorbed at 3.9 Å from graphene (bottom) are displayed with details of the solvation shells (insets). The hydroxide, hydrogen, boron, carbon, nitrogen and oxygen atoms are respectively represented in cyan, orange, grey, blue, and red

The resulting PMF curves are shown in Fig. 2a for h-BN (top) and graphene (bottom) as a function of $D_{{\mathrm{O}}^ \ast - {\mathrm{A}}^ \ast }$ together with statistical error bars. While both free energy profiles yield positive surface excesses of hydroxides (see Supplementary Methods), there are striking differences between the two interfaces.

For h-BN two minima are obtained: the first minimum is a stiff well at 1.5 Å lying at −0.15 ± 0.19 eV corresponding to a chemisorbed state predicted earlier^8^ and in agreement with numerous examples of covalent hydroxylation of h-BN^26^. The second minimum is a shallow one at 3.5 Å lying at −0.21 ± 0.13 eV corresponding to a physisorbed state. The two adsorption states are separated by a free energy barrier of 0.79 ± 0.17 eV.

For the graphene surface, there is a large repulsion wall at close distance in line with the earlier DFT study^8^ and a physisorbed state at 3.3 Å and −0.27 ± 0.13 eV. Interestingly the superposition of the water density on the PMF plots (see Supplementary Fig. 4) demonstrates that the first physisorption well on graphene matches the closest water layer while the chemisorption well on h-BN lies beneath this first layer. An atomistic snapshot of the anion in both states illustrates one instant of the dynamical solvated picture: in Fig. 2b top panel, the hydroxide anion is chemically bonded to a boron atom and is at the centre of a tetrahedron formed by a covalently bonded boron atom and three higher-lying water molecules—two hydrogen bond donors and one hydrogen bond acceptor. In Fig. 2b bottom panel, the hydroxide anion lies at the centre of a square planar complex formed by four H-bond donor water molecules lying in the first layer above graphene. A fifth water molecule lying further at the upper apex can also dynamically enter the solvation shell, thus forming a square pyramidal environment. In the latter solvation structure close to graphene, the hydroxide is greatly stabilized by around 11 *k*_B_*T*.

### Free MD runs

**Fig. 3:**
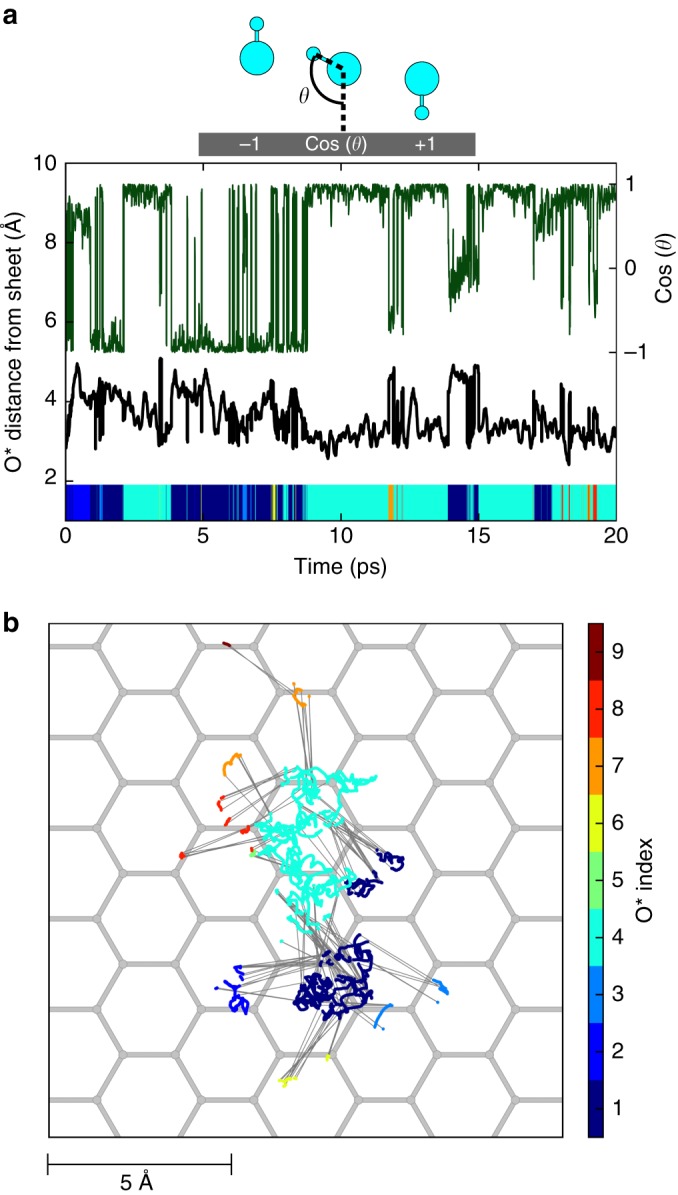
Unbiased displacements of OH^−^ on graphene. Time variations of the vertical displacement (**a**, black curve) and lateral displacement (**b**, coloured trajectories) of a free hydroxide starting at the physisorption distance from a graphene sheet. The cosine of the hydroxide orientation with respect to the surface is also plotted vs. time (**a**, dark green curve). The index of the hydroxide oxygen O^*^ is associated to a specific colour so that changes of colour represent successive proton transfers in time and hence hydroxide diffusion. Proton transfers are also indicated by thin grey straight lines on top of the carbon hexagonal grid represented in grey (**b**)

Our computed PMFs infer a fresh view of the graphene and h-BN interfaces in water and we now question the stability of the physisorption structures after removing all constraints. The resulting bias-free MD runs of 20 ps are visible in Supplementary Movies 1 and 2. Figure 3a shows the corresponding time evolution of the distance of the hydroxide from the surface (black curve): the O^*^ lies mainly around 3 Å away from the sheet with some residence time at 4 Å. In Fig. 3b the lateral displacement—*x*,*y* trajectory—of the hydroxide is displayed with colour codes corresponding to the changing index of the O^*^ atom. It can be seen that the hydroxide is not fixed in the *xy* plane and diffuses through proton transfers. The colour range permits to evaluate the average number of proton transfers (about 10 in 20 ps time). For comparison, the displacements both in vertical and lateral directions of the physisorbed hydroxide on the h-BN surface vs. time have been computed and show similar behaviour (see Supplementary Fig. 5). The self-diffusion coefficients of the hydroxide were derived from those trajectories by applying the Einstein equation with computed mean square displacements (see Supplementary Methods). Both the lateral and the vertical diffusions of the anion in the vicinity of the surfaces were determined. A hydroxide in bulk water was also simulated as a reference system to compare with the interfaces, using the same DFT dynamics framework. The isodirectional coefficient of a hydroxide in bulk water was obtained from a 50 ps MD trajectory. The obtained values are in qualitative agreement with both experimental and simulation data from the literature (see Table 1)^18,27,28^. Interestingly, although the diffusion of OH^−^ at the h-BN/water and graphene/water interfaces is vertically confined, its 2D motion remains in the same order of magnitude of that of the anion in bulk water, corroborating the picture of a mobile ion physisorbed on the hexagonal layers.

**Table 1:** Computed and experimental diffusion coefficients of OH^−^ in various media and conditions (units: 10^−5^cm^2^.s^−1^)

Medium	Conditions	$D^{//}$	$D^ \bot$	*D* ^iso^
h-BN	PBE-D3	**7.10** ± **1.62**	**0.46** ± **0.65**	
Graphene	PBE-D3	**12.00** ± **3.95**	**0.07** ± **0.11**	
D_2_O	PBE-D3			**4.46** ± **1.31**
D_2_O	PBE			18.2 ± 3.7^18^
D_2_O	PBE-TS			8.3 ± 1.6^18^
D_2_O	PBE0-TS			3.7 ± 0.4^18^
H_2_O	Exp.			5.4^19,28^, 5.2^19,29^
D_2_O	Exp.			3.2^19,28^, 3.1^19,29^

$D^{//}$ and $D^ \bot$ respectively correspond to lateral and vertical diffusion with respect to the surface, while *D*^iso^ refers to isodirectional diffusion. Values obtained in the present work are written in bold font and the corresponding errors (standard deviations) were estimated by block analysis of linear regressions over mean square displacements of the anion.

How to understand that fast proton transfer do not allow for hydroxide diffusion away from the graphene interface? The answer arises from the existence of hydrogen bonds within the first water layer as observed at water-vapour interfaces^29^ and from a particular orientation of the hydroxide at the graphene–water interface. Indeed in Fig. 3a the cosine vs. time of the hydroxide tilt angle with respect to the surface (green curve) shows that the hydroxide can adopt two orientations with cosine values close to −1 and +1, corresponding to two H-up (outwards the interface) or H-down (inwards the interface) conformations respectively. Notably in the H-down state -corresponding to cosine equals 1- the oxygen lies closer to the interface than in the H-up state (see Fig. 3a and Supplementary Movie 2).

When a hydroxide is lying H-down in close vicinity to the graphene, it can accept a proton from a H-bond donor water molecule lying above the anion but still remaining in the first water layer. After the proton transfer, the hydroxide ion is then found H-up and lying further away from the graphene sheet so that it can only accept protons from water molecules below, closer to the surface, but not from water molecules in the second layer from the surface. As a result, the hydroxide diffuses rapidly via flip-flop moves within the hydrogen bond network of the first water layer and its transfer to the second water layer is kinetically hindered. Note that in the case of h-BN, we find a slightly different picture with a smoother distribution of cosine angles enhancing the number of proton transfers by a factor of two during the 50 ps MD trajectory (see Supplementary Fig. 5).

**Fig. 4:**
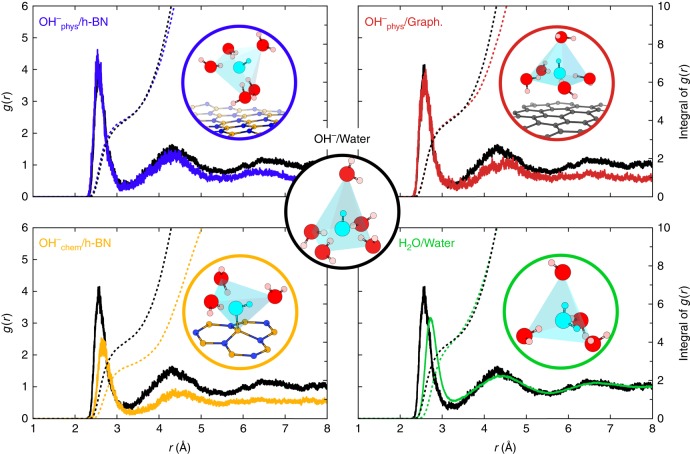
Radial distribution functions from unbiased trajectories. Radial distribution functions of oxygen atoms around the free hydroxide oxygen O* in different environments: bulk water (black), chemisorbed on h-BN (orange), physisorbed on h-BN (blue) and physisorbed on graphene (red). The *g*(*r*) of a H_2_O molecule in bulk water is shown in green. The integrals of the *g*(*r*) are displayed as dotted line in the corresponding colour. Solvation geometries of the different configurations are shown within circles of the associated colour: square pyramid for the anion in bulk water (black) or physisorbed on h-BN (blue) or on graphene (red), tetrahedron for a chemisorbed OH^−^ on h-BN (orange) or for a bulk water molecule (green)

It is also interesting to compare the solvation picture of the hydroxide ion described above to other environments. Figure 4 compares the radial distribution functions for physisorbed hydroxide on h-BN (blue), chemisorbed hydroxide on h-BN (orange), physisorbed hydroxide on graphene (red), hydroxide in bulk water (black) and H_2_O molecule in bulk water (green). Integrals of *g*(*r*) are superposed with dashed lines to quantify the number of water molecules in the solvation shells. Our results for the bulk systems agree well with the literature: the hydroxide (water) is found five-fold (four-fold) coordinated in a square-planar pyramid (tetrahedral) geometry in bulk water. The integral of the first peak of the *g*(*r*) for all cases except the chemisorption case reaches a plateau value of about 4 which corresponds to the number of nearest neighbour water molecules arranged in either the square planar or the tetrahedral geometry. It should be noted that the fifth apex water molecule of the pyramidal structure visible in three panels (blue, red and black) is not reflected in this plateau value demonstrating that it is only dynamically present, as further illustrated in Supplementary Fig. 6. Also, this integral of *g*(*r*) reaches 3 in the chemisorption case (orange dashed line), illustrating the partial desolvation of the hydroxide covalently bonded to the h-BN layer. Moreover, we observed that the diffusion mechanism for the physisorbed anion mediated by proton transfer is similar to that in bulk water^12^ albeit restrained to occur only within the first interfacial water layer. Once the anion is in the active 4-fold solvation configuration (receiving three H-bonds), frequent proton transfers occur through the standard tetrahedral water network, until OH^−^ finds itself once again in an inactive 5-fold environment (receiving four H-bonds).

### Comparison to conductance measurements in a carbon nanotube

These results show that not only the graphene surface can be charged through physisorption of hydroxide but also that there is a concomitant appearance of a surface conductivity through hydroxide transport within the first water layer. To confirm this picture, we now compare our findings with the experimental conductance of single carbon nanotubes (CNT) measured by Secchi et al.^4^. These authors measured the conductance of single CNTs passing through a membrane separating two compartments containing a solution of KCl at concentration *C*_S_ and at a pH fixed by addition of KOH. Since the tubes’s diameters range between 3.5 nm and 35 nm, the curvature effect remains subtle. Hence our simulated planar surfaces confining water in a space of around 2 nm width are appropriate atomistic models of the measured tubes.

In order to compare our results with experiments, it is necessary to take into account the electrostatic repulsion between adsorbed hydroxide ions and the presence of counterions in the double layer of the surface for charge equalization. These effects are missing in our simulation of a single hydroxide ion in absence of any counterion. Such effects however have been modelled successfully using mean-field approaches^30^.

Here, for the sake of comparison, we employ the same charge equalization model as in reference ^4^. We start by simplifying the PMF as a box potential of depth *U*_ads_ and width *d* (see Supplementary Discussion). Then, taking into account the electrostatic potential at the surface generated by a finite ion concentration, the chemical potential $\mu _{{\mathrm{OH}}^ - }$ of OH^−^ adsorbed at the graphene surface, for a given hydroxide surface concentration Σ, is

1 $$\mu _{{\mathrm{OH}}^ - } = k_{\mathrm{B}}T\log \left( {\frac{{\Sigma \Lambda ^3}}{d}} \right) - {\mathrm{e}}V_{\mathrm{S}} + U_{{\mathrm{ads}}},$$

where *k*_B_ is the Boltzmann constant and Λ is the OH^−^ de Broglie thermal wavelength at temperature *T* = 300 K. *V*_S_ is the electrostatic potential at the surface, which self consistently depends on the salt concentration and the surface charge itself. It was evaluated in ref. ^4^ using the non-linear Poisson-Boltzmann equation, leading to a power-law dependence of the surface charge on salt concentration at low ionic strength, which has been observed experimentally.

The conductance of the CNT is related to the surface concentration of hydroxide through the Poisson-Nernst-Planck (PNP) equation adapted to confined geometry. However, the usual picture for the conductance assumes that the adsorbed charges are immobile. Here, we contradict this picture, showing that the physisorbed hydroxide retains a large surface mobility. We evaluate the contribution of the mobile surface charge by first noting that the surface charge per unit length of a CNT of radius *R* is 2eπ*R*Σ, e being the elementary charge. The adsorbed ions have a mobility $\lambda _{{\mathrm{OH}}^ - }$, thus when submitted to an electric field *U*/*L*, where *L* is the CNT length and *U* the potential applied across the CNT, they acquire a velocity $v = \lambda _{{\mathrm{OH}}^ - }{\mathrm{e}}U/L$. In the following, we will take the experimental bulk mobility for hydroxide (see Supplementary Discussion) since we found in our simulation a similar mobility for physisorbed hydroxide or for bulk hydroxide. This leads to a contribution of the mobile surface charge to the conductance being equal to $G_{{\mathrm{Surf}}} = 2{\mathrm{e}}^2\lambda _{{\mathrm{OH}}^ - }\frac{{{\mathrm{\pi }}R}}{L}\Sigma$.

**Fig. 5:**
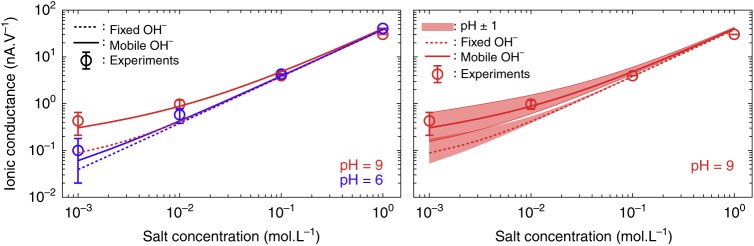
Ionic conductance, experiments vs. analytic model. (left) Ionic conductance *G* of a single 1500 nm long multiwall carbon nanotube with an inner diameter of 35 nm in function of the salt concentration at pH 6 (blue) and pH 9 (red). The circles correspond to experimental measurements *G* = *I*/Δ*V* (courtesy of Secchi et al.^4^). The dotted and plain curves are derived from analytic models respectively excluding and including the mobility of the hydroxide anion displayed here for comparison. (right) The effect of a variation of 1 pH unit on both models applied to the conductance at pH 9 is represented by coloured surfaces, with the upper (lower) bound corresponding to a pH increased (decreased) by 1 unit

We thus correct the usual PNP result for the conductance, *G*, with the hydroxide mobility, such that we write

2 $$G = 2{\mathrm{e}}^2\lambda \frac{{{\mathrm{\pi }}R^2}}{L}\sqrt {C_{\mathrm{S}}^2 + \frac{{\Sigma ^2}}{{R^2}}} + 2{\mathrm{e}}^2\lambda _{{\mathrm{OH}}^ - }\frac{{{\mathrm{\pi }}R}}{L}\Sigma ,$$

where *C*_S_ is the salt (KCl) concentration. $\lambda = \frac{1}{2}\left( {\lambda _{{\mathrm{Cl}}^ - } + \lambda _{{\mathrm{K}}^ + }} \right)$ is the KCl mobility. The first term thus accounts for the KCl contribution to the conductance, while the second term arises because of the mobile adsorbed charges. If we take *U*_ads_ = −400 meV, on the high side but within error bars of our predicted value, and *d* = 0.21 Å, as extracted from our PMF (see Supplementary Methods), we obtain results in quantitative agreement with experiments for the conductance of a CNT with *R* = 35 nm and *L* = 1500 nm, at pH = 6 and pH = 9, see Fig. 5 left panel. The model reproduces well the pH dependence of the conductance at low salt concentration, coherent with hydroxide ions being adsorbed on the CNT surface.

The importance of the mobility of the surface charges is clear from Fig. 5: the conductance predicted by omitting it is shown to deviate significantly from the experiments (see dashed lines in Fig. 5). For example, the predicted surface charge for a KCl concentration of *C*_S_ = 0.001 M and pH = 9 is eΣ = 0.01 C.m^−2^; it would lead to a conductance *G* = 0.1 nA.V^−1^ for the same CNT as above with fixed adsorbed charges, nonetheless it is four times larger, *G* = 0.4 nA.V^−1^, when taking into account the surface charge mobility. We have checked the stability of the predicted conductance with respect to variations in the parameters of the models. The effects of variations in *R* or *L* are limited (see Supplementary Discussion), while it is the largest for variations in pH. The effect of a pH variation of one unit is displayed in Fig. 5 right panel for the two models, including or not the surface charge mobility. The two predictions are still clearly separated showing that the effect of surface charge mobility is significant even with respect to variations of other parameters in the model. The effect of variations of the geometry of the tube are shown in Supplementary Figure 7.

Taking into account the surface charge mobility thus turns out to be crucial to interpret electrohydrodynamics phenomena at graphene surfaces. This mobility of adsorbed charges also affects other phenomena: Maduar et al.^31^ have discussed the large influence of surface charge mobility on the Zeta potential. Here, we thus give a novel microscopic explanation as to why graphene is a good material candidate to exploit nanoscale transport phenomena such as electrophoresis, and electrosmosis, for blue energy production or water desalination^4,6^.

## Discussion

In summary, we have shown by means of ab initio molecular dynamics that graphene and h-BN single layers can both charge up in contact with alkaline water but in a different way. On a graphene surface the hydrated hydroxide anion is physisorbed albeit diffusing laterally via lateral proton hops. On h-BN surface either the hydroxide anion covalently binds to a boron surface atom or remains physisorbed and mobile. These findings originate from free energy profiles of one OH^−^ near the surfaces and have been further confirmed by unbiased MD trajectories starting at the PMF adsorption wells. Analytic developments accounting for the unveiled surface mobility of the adsorbed charges are very consistent with ionic conductance measurements performed earlier in nanofluidic devices. Understanding the atomistic mechanism behind tremendous surface charges measured on graphene and boron nitride nanomaterials in mild aqueous conditions is promising to identify industrially relevant candidate materials for blue energy production and water desalination applications^6^.

This charging mechanism can possibly be further investigated experimentally at the molecular scale by means of sum-frequency generation spectroscopy as the OH^−^ remains mainly in the vicinity of the graphene surface. Also, the confinement of the hydroxide at the interface, with proton transfer restrained to the first interfacial water layer raises the question of its validity in extremely confined channels such as the recently elaborated ångstrom slit-pores nanofluidic devices^32,33^. In fact, only up to two water layers can penetrate in between the graphite layers constituting those channels. As interfacial molecular information on such system is not accessible experimentally, this will be the object of future AIMD studies, in the same spirit of the recent hydroxide solvation modelling in inorganic mackinawite slit-pores^34^. Slit-pores channels made of non-metallic materials could also provide an appropriate substrate to probe the normal orientation of the hydroxide to the surfaces by the above-mentioned interfacial spectroscopy technique.

## Methods

### Computational Details

AIMD was performed with the CP2K code^35^. The computations of the forces were carried out using the implementation of the DFT of the QuickStep module^36,37^. DZVP-MOLOPT-SR-GTH basis sets were used^38^ along with plane waves expanded to a 600 Ry energy cutoff. Electronic cores were represented by Geodecker-Teter-Hutter pseudopotentials^39–41^. The Perdew, Burke and Ernzerhof (PBE) functional was used^42^ with the D3 dispersion correction scheme^43,44^. PBE-D3 has indeed been recently shown to provide a good description of the water/graphene interface^45^.

The h-BN (graphene) single layer/water interface was modelled using a 13.04 Å × 12.55 Å × 21.0 Å (12.83 Å × 12.35 Å × 21.0 Å) orthorhombic cell containing 60 surface atoms arranged in a hexagonal monolayer, one hydroxide anion and 97 (94) water molecules under periodic boundary conditions. This corresponds to surfaces separated by a ~ 16 Å thick and 0.57 M hydroxide aqueous solution yielding pH 13.8. Simulations in bulk water were performed in a 12.42 Å side cubic cell containing 63 water molecules and one charged hydroxide. The deuterium mass is substituted for all protons to reduce the time step size needed for energy conservation in our Born-Oppenheimer AIMD and to limit nuclear quantum effects. The anion containing simulation cells did not include a counterion and the systems therefore presented a net charge neutralized by a uniformly charged background. AIMD simulations were carried out with a 0.5 fs timestep in the NVT ensemble at 323 K using Nose-Hoover thermostats^46,47^ with a time constant of 500 fs. At this increased temperature condition the PBE functional predicts a reasonable liquid water structure. The convergence of the WHAM procedure is illustrated in Supplementary Fig. 8. More information regarding computational details as well as a typical CP2K input file are respectively included in Supplementary Methods and Supplementary Note 1.

## Supplementary information

Supplementary Information

Peer Review File

Description of Additional Supplementary Files

Supplementary Movie 1

Supplementary Movie 2

Supplementary Movie 3

## Data Availability

The source data underlying Figs 1b, 2a, 3, 4 and 5 are accessible as source data files (https://figshare.com/articles/Plots_source_data/7623953) along with a corresponding python notebook (https://figshare.com/articles/Plots_python_notebook/762397). Experimental data points displayed Fig. 5 has been provided by Secchi et al.^4^. Other data is available upon reasonable request to the authors.
