# Supplementary material for: Versatile electrification of two-dimensional nanomaterials in water
Source: Nat Commun. 2019 Apr 10;10:1656. doi: 10.1038/s41467-019-09708-7 (PMC6458114; doi:10.1038/s41467-019-09708-7)
Supplement: Supplementary file 1 — Supplementary Information [file 41467_2019_9708_MOESM1_ESM.pdf]

Supplementary Information to:
"Versatile Electrification of Two-dimensional Nanomaterials in Water"

Benoît Grosjean *et al.*

SUPPLEMENTARY METHODS

All simulations were performed with a 0.5 fs timestep in the NVT ensemble with the CP2K 3.0 code [1–7]. DZVP-MOLOPT-SR-GTH basis sets [8] were used alongside planewaves expanded to a 600 Ry energy cutoff. Electronic cores were represented by Geodecker-Teter-Hutter pseudopotentials [9–11]. The Perdew, Burke and Ernzerhof (PBE) functional [12] was used with the D3 dispersion correction scheme [13, 14]. The temperature was set to 323.15 K by means of a Nose-Hoover thermostat [15, 16] with a time constant of 500 fs. An example of a CP2K input file for the coordination restrained trajectories is available at the end of the present document (see Supplementary Note 1).

Simulation protocol for the free energy profiles

The potential of mean force of the aqueous hydroxide anion with respect to its distance to a hBN or graphene layer is obtained by thermodynamic integration over the component normal to the surface of the force acting on the oxygen O^* of OH^-, $F^{\perp}_{O^*}$ (see below). To obtain $\left\langle F^{\perp}_{O^*} \right\rangle_r$, the mean force of interest for a surface-anion distance r, molecular dynamics are performed with constraining this distance by freezing a surface atom A^* and the hydroxide oxygen O^*. Starting close to the material, the anion is progressively displaced until it reaches 6 Å. Trajectories are computed for each value thereby spanned by r. However, this requires to keep track of the elusive anion prone to proton exchange with solvating water molecules. To prevent proton hops, the hydrogen coordination of the hydroxide oxygen n_{O^*-H} is restrained around a target value n^* by applying an harmonic potential W of the form:

$$W = k(n_{O^*-H} - n^*) \tag{1}$$

with $k = 0.05$ Ha a force constant and

$$n_{O^*-H} = \sum_{i=1}^{N} \frac{1 - (\frac{r_i}{R_0})^{12}}{1 - (\frac{r_i}{R_0})^{20}} \tag{2}$$

where i runs over every hydrogen atom of the simulation box (N in total); with r_i the distance between hydrogen i and O^*. R_0 is a switch distance set to 1.2 Å which corresponds to the minimum between the first and second peak of the radial distribution function $g(r)$

of hydrogen atoms around O^* computed for a free OH^- in bulk water. This is illustrated in Supp. Fig. 1 in which the $g(r)$ is displayed in black and the switch function (see Eq. 2) in blue. Because of the dependency of n_{O^*-H} on the first solvation shell (see Supp. Fig. 1, non-zero values of the blue curve between 1.2 and 2 Å), the hydrogen coordination target of the restraint n^* (see Eq. 2) does not necessarily correspond to the desired number of hydrogen atoms in the covalent shell of O^*. In fact, $\langle n_{O^*-H} \rangle$ varies along with $D_{O^*-A^*}$ and more generally with its environment, as illustrated in Supp. Fig. 2 in which histograms of n_{O^*-H} are displayed for different cases. One can observe a clear difference between the histogram of a free OH^- chemisorbed on h-BN (orange curve) and that of the free anion in bulk water (black curve). The latter exhibits a peak centered at 1.3 that corresponds to the standard picture of an anion isolated within a solvation shell. However a part of this distribution crosses the one of H_2O in bulk water (green curve) around 1.8 and that overlap corresponds to configurations where the hydroxide is in the form of a transient $H_3O_2^-$ species in which a proton is shared between the hydroxide and a water molecule (see the inset in the black circle). In this second situation, the coordination of the anion resembles that of a bulk water molecule (green curve) and proton transfer is prone to occur. We therefore wish to avoid those configurations, by centering the coordination distribution either on the peak of the chemisorbed anion (around 1.0) or on the peak of the standard solvated hydroxide (around 1.3). Configurations excluding proton transfer were therefore obtained, as illustrated by the distribution for the anion in bulk water with its coordination restrained to 1.3 (red curve). Since two coordination values were to be considered to represent the system in which the anion is either chemisorbed or in bulk water or in-between, two dynamics are performed for each finite value of $D_{O^*-A^*}$ with n^* respectively set to 1.0 and 1.3. For each distance probed, the MD runs are the object of a further analysis before computation of the PMF can be performed (see below).

Simulation cells and simulation procedure

For graphene (h-BN), the 12.83 Å × 12.35 Å × 21.0 Å (13.04 Å × 12.55 Å × 21.0 Å) starting orthorhombic simulation cell contained 96 (99) water molecules surrounding a 60 atoms single layer. The boxes were equilibrated for 5 ps before 50 ps production runs were performed, in particular to derive the water density profiles of Supp. Fig. 4 (blue

curves). A hydrogen atom was deleted in the last configuration of each trajectory to yield simulation cells containing a hydroxide anion. The hydroxide was fixed at a chemisorption distance of 1.5 Å from a surface atom by freezing the surface site and the hydrogen oxygen O^*. Its hydrogen coordination was restrained during an equilibration run of 5 ps. To probe the desired range of values for $D_{O^*-A^*}$, successive dynamics were performed with the hydroxide translated by 0.1 or 0.2 Å using the last atomic configuration of the previous run. For each distance, the system was equilibrated for 0.5 ps plus an additional 0.5 ps if restraint parameters were modified. For each value of $D_{O^*-A^*}$ and for each value of the restraint target, production runs were then performed. Two sets of MD trajectories have thus been computed with the restraint parameter n^* respectively set as 1.0 and 1.3 (see Eq. 1). Production runs of 2 to 3 ps were performed with the first target value and between 4 and 6 ps for the second one. Every configurations in which proton transfer occurred were excluded from data analysis.

Non-biased trajectories

The simulation of a free hydroxide in bulk water was performed on a 12.42 Å × 12.42 Å × 12.42 Å unit cell containing 63 water molecules and 1 OH^-. The system was equilibrated for 5 ps before a 50 ps production run was computed.

The last snapshot of the restrained trajectory with the appropriate restraint parameter and anion-surface distance was used as the starting atomic configuration for non-biased simulations. We started from pre-equilibrated systems at the considered distances: the 20 ps trajectory of a free physisorbed anion on graphene (h-BN) was computed starting from a configuration obtained with $D_{O^*-A^*}$ and n^* respectively equal to 3.2 Å and 1.3 (3.6 Å and 1.3). For the chemisorption case, $D_{O^*-A^*}$ and n^* were respectively equal to 1.5 Å and 1.0 for both graphene and BN. In the corresponding movies, the hydrogen, boron, carbon, nitrogen and oxygen atoms are respectively represented in white, orange, grey, blue and red while the hydroxide is displayed in cyan. These movies are a good illustration for the main features of the PMFs depicted in the main Fig. 2. In Movies 1 and 2, one can observe that although free, the hydroxide remains mainly within the water layer closest to the surface, as suggested by the physisorption wells of the PMFs when plotted along the water density (see Supp. Fig. 4). Regarding the significant behaviour difference at short distances, one

can observe in Supplementary Movie 3 that the hydroxide leaves the graphene layer within the first 500 fs of the trajectory while it sticks to the h-BN surface. Although not displayed in the animation, the anion remains covalently bonded to the h-BN layer for the whole 15 ps trajectory.

Similarly to main Fig. 3, the evolution of the vertical height and the horizontal displacement of the hydroxide on top of a BN layer is displayed in Supp. Fig. 5. It is noteworthy that the anion experiences about three times more proton transfers, exploring a surface roughly three times larger on h-BN than it does on graphene. The hydroxide explores tilt angles between 0 and 180° (dark green curve) contrary to the case of graphene for which the internuclear axis of the anion remains mostly perpendicular to the surface.

Simulations pressure

As the pressure component normal to the surface can play a role in adsorption properties, it was evaluated inside the simulation cells. The atomic forces were recomputed on 200 snapshots distant by 0.1 ps in the 20 ps free trajectories of the physisorbed hydroxide, after adding an additional 10 Å vacuum separation between water and one side only of the surface. The pressure was then evaluated as the average over those 200 snapshots of the sum of the normal component of the forces acting on the surface atoms divided by the simulation cell section area. Mean pressures of 0 ± 95 MPa and 73 ± 93 MPa were thereby derived for the h-BN and graphene simulation cells, respectively. The errors correspond to standard deviations derived by block analysis (see below). The overall structure of water was found unaffected by the slab geometry when comparing the radial distribution functions of the liquid between the interfacial systems and bulk water.

Movies

All movies correspond to non-biased dynamics: 20 ps run of a physisorbed OH^- on h-BN (Supplementary Movie 1), 20 ps run of a physisorbed OH^- on graphene (Supplementary Movie 2), and 500 fs runs of the chemisorbed anion on h-BN (Supplementary Movie 3, left) and graphene (Supplementary Movie 3, right). Movies were generated using the Visual Molecular Dynamics (VMD) software [17–19].

Weighted histogram analysis method

The two sets of computed trajectories were combined using the Weighted Histogram Analysis Method (WHAM) of Kumar *et al.* [20]. The extension of this method proposed by Souaille and Roux [21] was applied independently for each given value of $D_{\mathrm{O}^*-\mathrm{A}^*}$ to appropriately re-weight every snapshot of the two dynamics and combine them into a WHAM trajectory along which observables could be computed:

$$\langle A \rangle = \frac{1}{\sum_{i,l} w_{i,l}} \sum_{i=1}^{2} \sum_{l=1}^{N_i} w_{i,l} A_{i,l} \tag{3}$$

where A is an observable, i refers to one of the two trajectories of length N_i (both simulated at a given value of $D_{\mathrm{O}^*-\mathrm{A}^*}$), l indicates the lth snapshot of the ith trajectory and $w_{i,l}$ designates the weight obtained by WHAM for the lth snapshot of trajectory i. The two sets of MD trajectories can be visualized by the associated mean hydrogen coordination (see Supp. Fig. 8 a and b) while one can represent the WHAM trajectory with the WHAM derived mean hydrogen coordination $\langle n_{\mathrm{O}^*-\mathrm{H}} \rangle$, as displayed by white curves in Supp. Fig. 3.
To illustrate the convergence of the WHAM process, the overlap of the coordination histograms of the two recombined MD runs (see Supp. Fig. 8c) for each distance is displayed in Supp. Fig. 8 a and b by representing the histograms vertically with the associated probabilities visualized by color scales.

Thermodynamic integration

Once the trajectories recombined along the hydrogen coordination dimension by WHAM, the average $\left\langle F^{\perp}_{\mathrm{O}^*} \right\rangle_r$ of the component normal to the surface of the force acting on O^* was computed for every given value r of $D_{\mathrm{O}^*-\mathrm{A}^*}$ using Eq. 4. These averages were then integrated to yield the free energy of OH^- at a distance d from the surface as a potential of mean force (PMF), taking as reference the situation in which the anion is at the furthest distance from the surface.

$$\mathrm{PMF}(d) = \int_{\mathrm{bulk}}^{d} \left\langle F^{\perp}_{\mathrm{O}^*} \right\rangle_r dr \tag{4}$$

The overall PMF computation strategy is illustrated in Supp. Fig. 3: the anion-surface distance was probed by blue moon sampling as this variable was fixed during each MD run

(blue arrow). To perform umbrella sampling along the hydrogen coordination dimension (white arrow), two sets of trajectories with different restraint targets were computed (two white horizontal lines). For each given anion-surface distance, the two trajectories were recombined using WHAM allowing for the computation of the relevant mean forces on which thermodynamic integration was performed (red arrow). The WHAM trajectory is represented by the mean hydrogen coordination displayed as a white curve.

Estimation of errors

The error on observables computed as statistical averages along the trajectory were derived by artificially reducing the overall data correlation as we resorted to block analysis which consists in dividing the trajectory into a varying number of blocks n_{b} of identical length [22]. Once the average of each block is computed, the mean of all the block averages is derived alongside its standard deviation σ_{blocks}. An estimation of the deviation on the trajectory σ_{traj} can be obtained by linear regression as it can be approximately linked with σ_{blocks} and n_{b} in the following relation:

$$\sigma_{\mathrm{blocks}}^2 \approx \sigma_{\mathrm{traj}}^2 \times (n_{\mathrm{b}} - 1) \tag{5}$$

In the case of observables computed on the WHAM trajectory, we considered blocks of identical total WHAM weight instead of identical snapshot length.

To estimate the standard deviation $\sigma_{\mathrm{PMF}}(i)$ of the ith point of the PMF, the square of the deviations σ_r of $\left\langle F_{\mathrm{O}^*}^{\perp} \right\rangle_r$ obtained for each intermediate distance r were summed:

$$\sigma_{\mathrm{PMF}}(i)^2 = \sum_{k=1}^{i} \left(\sigma_{r_k} \frac{r_k - r_{k+1}}{2} \right)^2 \tag{6}$$

where $r_{k=1}$ corresponds to the furthest distance from the surface. The deviations σ_{r_k} were obtained using the above-mentioned WHAM corrected block analysis.

Derivation of free energy surfaces

Combining the PMF obtained by thermodynamic integration with $P(n|r)$ the coordination probability distributions for a given value r of $D_{\mathrm{O}^*-\mathrm{A}^*}$, one can reconstruct two-dimensional PMFs as displayed in Supp. Fig. 3 and 6:

$$\text{PMF}(n,r) = -k_\text{B}Tln(P(n,r)) + \alpha \tag{7}$$

where $T = 323$ K, k_B is the Boltzmann constant, α is a constant, n refers to the coordination and P to probabilities. The probability to find the system at (n, r) is obtained as follows:

$$P(n,r) = P(n|r).P(r) \tag{8}$$

where $P(r)$ is directly derived from the one-dimensional PMF described above:

$$P(r) = C.e^{-\frac{1}{k_\text{B}T}\text{PMF}(r)} \tag{9}$$

where C is a constant that vanishes along with α upon normalization of the total probability. The energy surface thus obtained supports the necessity of probing the coordination of the hydroxide as a reaction variable. In fact, one can observe on the upper map of Supp. Fig. 3 that the two energy minima of the case of h-BN are found at significantly different coordination values.

Diffusion coefficients

The diffusion coefficients were computed using the Einstein equation with the mean square displacements (MSD) derived from the unbiased MD trajectories:

$$\frac{1}{2d}\left\langle X^2(t)\right\rangle = Dt \tag{10}$$

where d is the dimensionality of the displacement X, D is the diffusion coefficient and t designates time. The mean displacement of N_part particles along a t_max long trajectory is obtained as follows:

$$\left\langle X^2(t)\right\rangle = \frac{1}{N_\text{part}}\sum_{i=1}^{N_\text{part}}\sum_{t=0}^{t_\text{max}}\frac{1}{n_\tau}\sum_{\tau=0}^{t_\text{max}-t}\left(X_i(\tau) - X_i(\tau+t)\right)^2 \tag{11}$$

where n_τ is the number of snapshots between 0 and $t_\text{max} - t$. The diffusion coefficients were obtained by linear regression over equation (6). The coefficient for H_2O molecules in bulk water was found to be $0.49 \pm 0.15\ 10^{-5}.\text{cm}^2.\text{s}^{-1}$, in the range of the literature values derived by AIMD [23, 24]. In that case, the length of the trajectory (50 ps) and the number

of particles considered (64) yielded a statistic large enough to compute the coefficient by dividing the trajectory into five blocks of 10 ps each. A value was derived on each block by linear regression between 0.3 and 10 ps. The final coefficient is an average of the 5 thus derived values with the corresponding standard deviation taken as the error. Nonetheless a different procedure was adopted for the diffusion of the hydroxide since the MSD of only one particle is considered, yielding a too small statistic to relevantly divide the data into blocks. Linear regressions on the MSD were performed over a 5 ps interval sliding between 0.3 and 45 ps for OH^- in water and between 1 and 15 ps for the anion close to the surfaces. The diffusion coefficients were averaged over all these regressions and the corresponding standard deviation was taken as an estimate of the error.

Gibbs dividing surface

The derivation of the position of the Gibbs dividing surface (GDS), r_{GDS} was adapted from Bonthuis *et al.* [25], considering the interface between water and the atomic surface at which the liquid density is canceled out:

$$r_{\mathrm{GDS}} = \int_0^{r_{\mathrm{bulk}}} \left(1 - \frac{\rho_{\mathrm{H_2O}}(r)}{\rho_{\mathrm{H_2O}}(r_{\mathrm{bulk}})}\right) dr \tag{12}$$

where r designates the distance to the surface and $\rho_{\mathrm{H_2O}}$ is the water density corresponding to the blue curves in Supp. Fig. 4. The average of the density profiles between 5 Å and the furthest point from the surface (10.5 Å) is taken as the bulk value allowing to define r_{bulk} as the furthest distance from the interface for which this density value is reached. r_{GDS} yielded 1.8 Å for both graphene and h-BN.

Surface excess

Adapting from the expression of Horinek and Netz [26], the surface excess of hydroxide Γ was derived as

$$\Gamma = [\mathrm{OH^-}]_{\mathrm{bulk}} \int_{1.45\mathring{A}}^{5.9\mathring{A}} \left(e^{-\frac{\mathrm{PMF}(r)}{k_{\mathrm{B}}T}} - 1\right) dr \tag{13}$$

where $T = 300$ K, $[\mathrm{OH^-}]_{\mathrm{bulk}} = 0.57$ M (corresponding to pH = 13.8) is the bulk concentration of hydroxide extrapolated for water in ambient conditions considering the molecular ratios

of OH^- to H_2O molecules in the simulation cells. Positive surface excesses of 38.5 $C.m^{-2}$ (399 $\mu mol.m^{-2}$) and 6.2 $C.m^{-2}$ (64 $\mu mol.m^{-2}$) were respectively obtained for graphene and h-BN. The surface charges thus derived are higher than experimental values [27, 28] since Eq. 13 corresponds to the case of infinite dilution with no counter-ions, contrary to the analytic model of the ionic conductance reported here.

SUPPLEMENTARY DISCUSSION

Observations regarding the solvation shell

Although n_{O^*-H} is meant to probe the covalent shell of O^*, because of its smoothness the switch function accounts for additional contributions of hydrogen atoms of the first solvation shell (see Supp. Fig. 1, non-zero values of the blue curve between 1.2 and 2 Å). This yields non-integer values of n_{O^*-H} comprised between 0.9 and 2 that can not be used for clear observations regarding the solvation shell of the hydroxide beyond identifying an overall increase or decrease of the solvation of the anion.

To allow clearer observations on the solvation shell, similarly to n_{O^*-H}, one can define the oxygen coordination of O^*, n_{O^*-O}, with the R_0 parameter set to 3.2 Å (see Eq. 2), corresponding to the minimum between the second and third peak of the radial distribution function $g(r)$ of oxygen atoms around O^* computed for a free OH^- in bulk water. The 12 and 20 exponents in Eq. 2 were respectively switched to 40 and 100 to yield a coarser function allowing for a clearer physical interpretation of n_{O^*-O}. The treatment described in Supplementary Methods was applied to n_{O^*-O} to map the free energy of the hydroxide with respect to $D_{O^*-A^*}$ and n_{O^*-O} (see Supp. Fig. 6). Contrary to n_{O^*-H}, n_{O^*-O} mainly probes the first solvation shell of OH^-, taking near-integer values corresponding roughly to the number of solvating water molecules. One can therefore observe that while physisorbed the hydroxide is mainly solvated by four water molecules (in a square planar geometry) but it can dynamically be surrounded by either three (when the square planar environment is distorted) or five solvating molecules (when a molecule approaches close enough in apex position of a square planar pyramid to be accounted in the oxygen coordination, see the red inset). While chemisorbed on h-BN, the oxygen coordination of the anion oscillates between two and three as the H-bond acceptor solvating molecule moves closer or further from the

hydroxide while two H-bond donors remain at short distances (see the orange inset).

Charge equilibration model

We start from the free energy of N adsorbed hydroxides on a graphene surface of area S at an electrostatic potential V_S:

$$F(N) = -k_{\mathrm{B}}T \log \frac{q^N}{N!} e^{-\frac{\mathrm{e}V_{\mathrm{S}}}{k_{\mathrm{B}}T}}, \tag{14}$$

where $T = 300$ K and q is the partition function for one hydroxide. Since the calculated PMF corresponds to averaging over all other degrees of freedom except the O^* translation, we may write

$$q = \frac{S}{\Lambda^3} \int_{r=0}^{r^*} e^{-\frac{U(r)}{k_{\mathrm{B}}T}}\, dr, \tag{15}$$

where Λ is the De Broglie thermal wavelength, $U(r)$ the PMF, and r^* a cutoff distance separating adsorbed hydroxide from bulk hydroxide. In doing so, we have assumed that the PMF $U(r)$ does not depend on the lateral position of the hydroxide, x, y, while we have computed $U(r)$ for a given x, y, at an apical position over a carbon atom of the graphene layer. This assumption is validated by the fact that we observe no corrugation effect in the free trajectory for an adsorbed hydroxide. For comparison with Secchi *et al.* [28] we further rewrite the one particle partition function as

$$q = \frac{Sd}{\Lambda^3} e^{-\frac{U_{\mathrm{ads}}}{k_{\mathrm{B}}T}}, \tag{16}$$

where U_{ads} is the bottom value of the PMF and the characteristic distance d is

$$d = \int_{r=0}^{r^*} e^{-\frac{(U(r)-U_{\mathrm{ads}})}{k_{\mathrm{B}}T}}\, dr. \tag{17}$$

We have computed d numerically taking $r^* = 5.9$ Å, the largest value of r for our calculated PMF, and found $d = 0.21$ Å. This value is nearly independent of r^* for $r^* > 4$ Å. It is then straightforward to obtain the chemical potential of adsorbed hydroxide ions:

$$\mu_{\mathrm{S}} = k_{\mathrm{B}}T \log\left(\frac{\Sigma\, \Lambda^3}{d}\right) - \mathrm{e}V_{\mathrm{S}} + U_{\mathrm{ads}}, \tag{18}$$

with $\Sigma = N/S$ the surface density of hydroxides.

In the reservoirs far from the carbon nanotube (CNT) the chemical potential of the hydroxide is simply

$$\mu_{\mathrm{B}} = k_{\mathrm{B}}T \log\left(\frac{[\mathrm{OH}^-]}{c_0} c_0 \mathcal{N}_{\mathrm{a}} \Lambda^3\right), \tag{19}$$

where $[\mathrm{OH}^-]$ is the hydroxide concentration in mol.L^{-1}, $\mathcal{N}_{\mathrm{a}}$ is the Avogadro number and c_0 the standard concentration $c_0 = 1$ mol.L^{-1}, such that $[\mathrm{OH}^-]c_0\mathcal{N}_{\mathrm{a}}$ is the hydroxide number density. At equilibrium $\mu_{\mathrm{S}} = \mu_{\mathrm{B}}$ and we find

$$\Sigma = \frac{10^{\mathrm{pH}}}{K_{\mathrm{e}}} c_0 \mathcal{N}_{\mathrm{a}} d e^{-\frac{U_{\mathrm{ads}}}{k_{\mathrm{B}}T}} e^{\frac{V_{\mathrm{S}}}{k_{\mathrm{B}}T}}, \tag{20}$$

where $K_{\mathrm{e}} = 10^{14}$ is the water dissociation constant. This equation is identical to the result of Secchi *et al.* with the quantities d and U_{ads} defined here from the calculated PMF. As V_{S} itself depends on the surface charge, this equation has to be solved self-consistently for Σ. We employ the same charge equalization model as Secchi *et al.* [28]. Finally, to compute the conductance of the CNT, we have considered the experimental mobility for KCl $= \frac{1}{2}(\lambda_{\mathrm{Cl}^-} + \lambda_{\mathrm{K}^+}) = 4.8 \times 10^{11}$ s.kg^{-1}. Similarly, we have considered the bulk mobility of OH^-, inferred from its diffusion constant, $\lambda_{\mathrm{OH}^-} = \frac{D_{\mathrm{OH}^-}}{k_{\mathrm{B}}T} = 13 \times 10^{11}$ s.kg^{-1}.

The fluctuations of analytic conductance derived by including or excluding the second term of Eq. 2 of the main text are displayed by colored surfaces in Supp. Fig. 7. Variations of 200 nm of tube length (a) and of 5 nm of tube radius (b) are displayed. It appears that the geometry of the system has a negligible effect on its conductance contrary to an increase of pH which displays a notable influence (see Fig. 5b of the main text).

SUPPLEMENTARY FIGURES

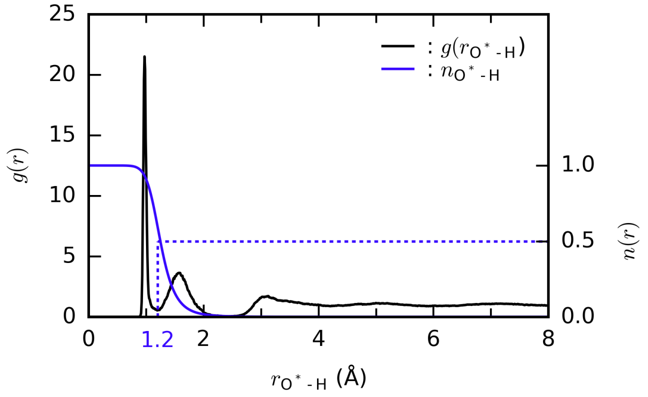

Supplementary Figure 1. The radial distribution function $g(r)$ of hydrogen atoms around the hydroxide oxygen O* is plotted in black. The corresponding contribution to the hydrogen coordination n_{O^*-H} is displayed in blue.

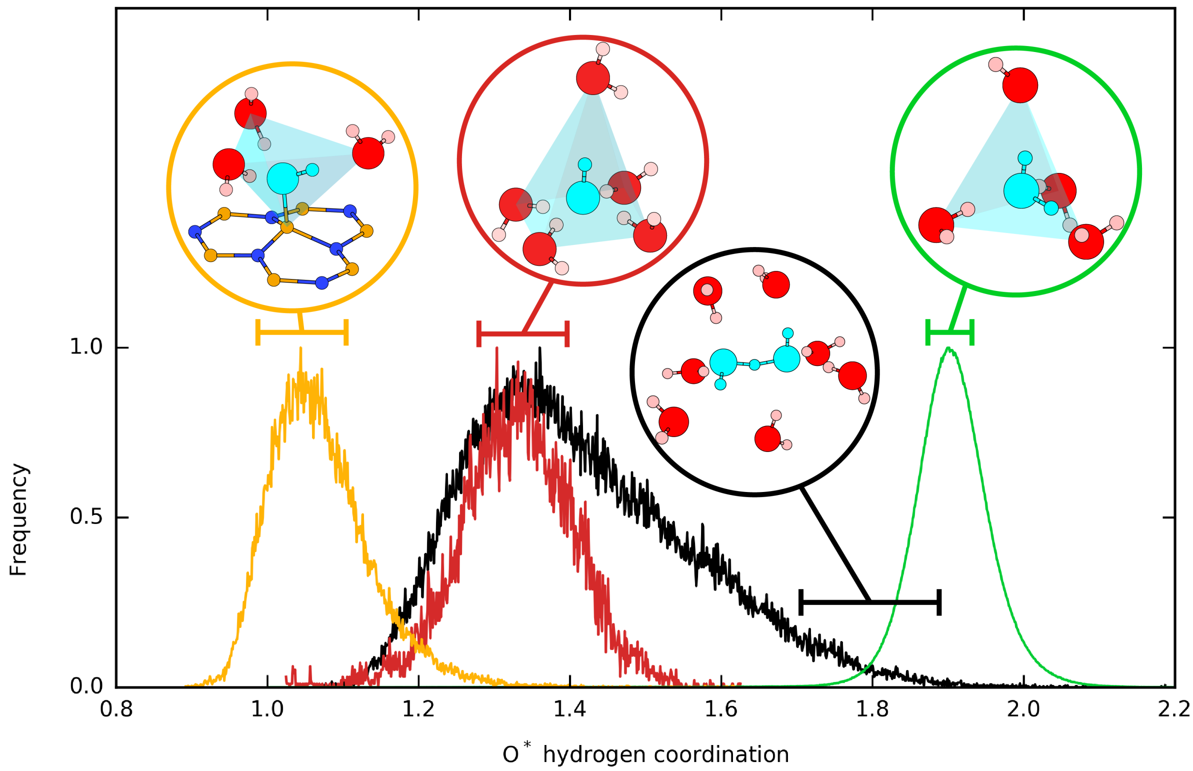

Supplementary Figure 2. Histograms of the hydrogen coordination of a free hydroxide chemisorbed on h-BN (orange line), of a free hydroxide in bulk water (black line), of a coordination restrained hydroxide in bulk water (red line) and of bulk water molecules (green line). Insets exhibit the solvation geometry of corresponding configurations. Oxygen and hydrogen atoms are respectively displayed in red and pink. The solvated species (H_2O, OH^- and $H_3O_2^-$) are represented in cyan. The unrestrained hydroxide oxygen O^* was identified as the closest oxygen atom to one hydrogen only. Each histogram was normalized so that its maximum would be 1.0.

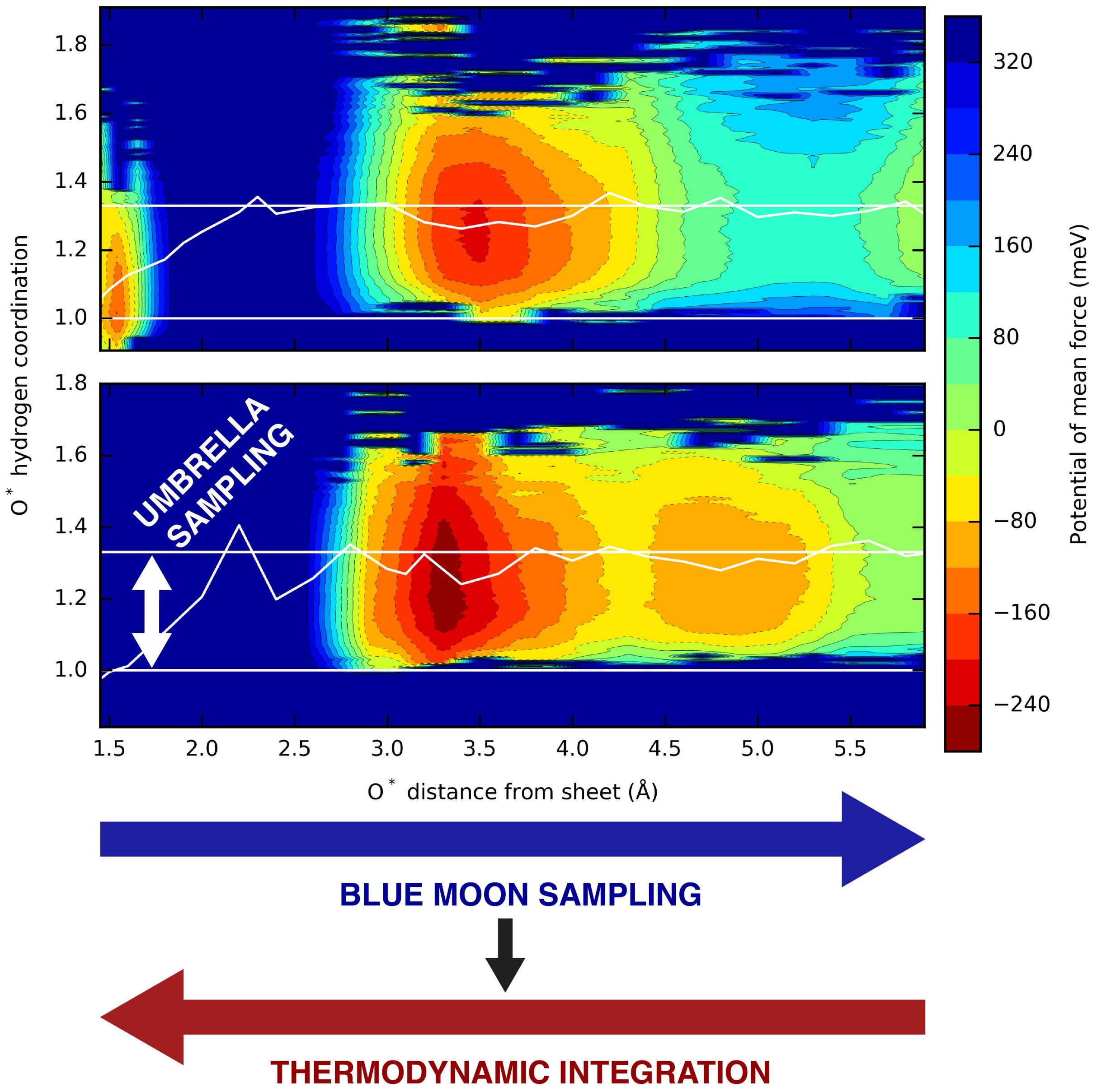

Supplementary Figure 3. Free energy profiles of OH^- with respect to its hydrogen coordination and its distance from a single layer h-BN (top) and graphene (bottom). White horizontal lines represent the two sets of MD trajectories, computed for a coordination target of 1.0 and 1.3. The WHAM mean hydrogen coordinations are displayed by white curve, thus representing the WHAM trajectory obtained by umbrella sampling between the two sets of dynamics. The blue arrow represents the direction of blue moon sampling - along the anion-surface distance - along which the thermodynamic integration (red arrow) is performed starting from the furthest distance from the surface.

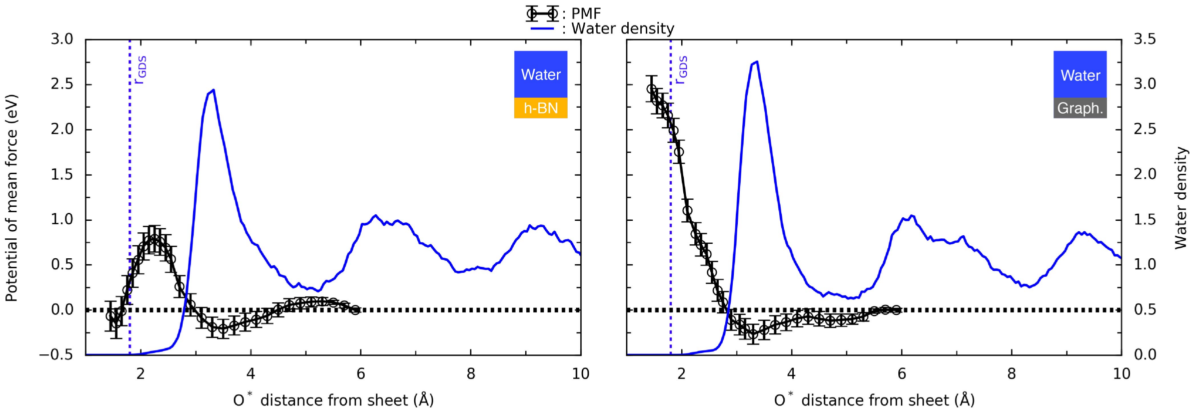

Supplementary Figure 4. Potentials of mean force (black lines) of an aqueous hydroxide $(O^{*}H)^{-}$ and water densities (blue lines) with respect to the O^{*} distance from the h-BN (left) and the graphene (right) sheets. The position of the Gibbs dividing surface (GDS) is indicated by vertical blue dotted lines. Error bars correspond to standard deviations (see Supplementary Methods)

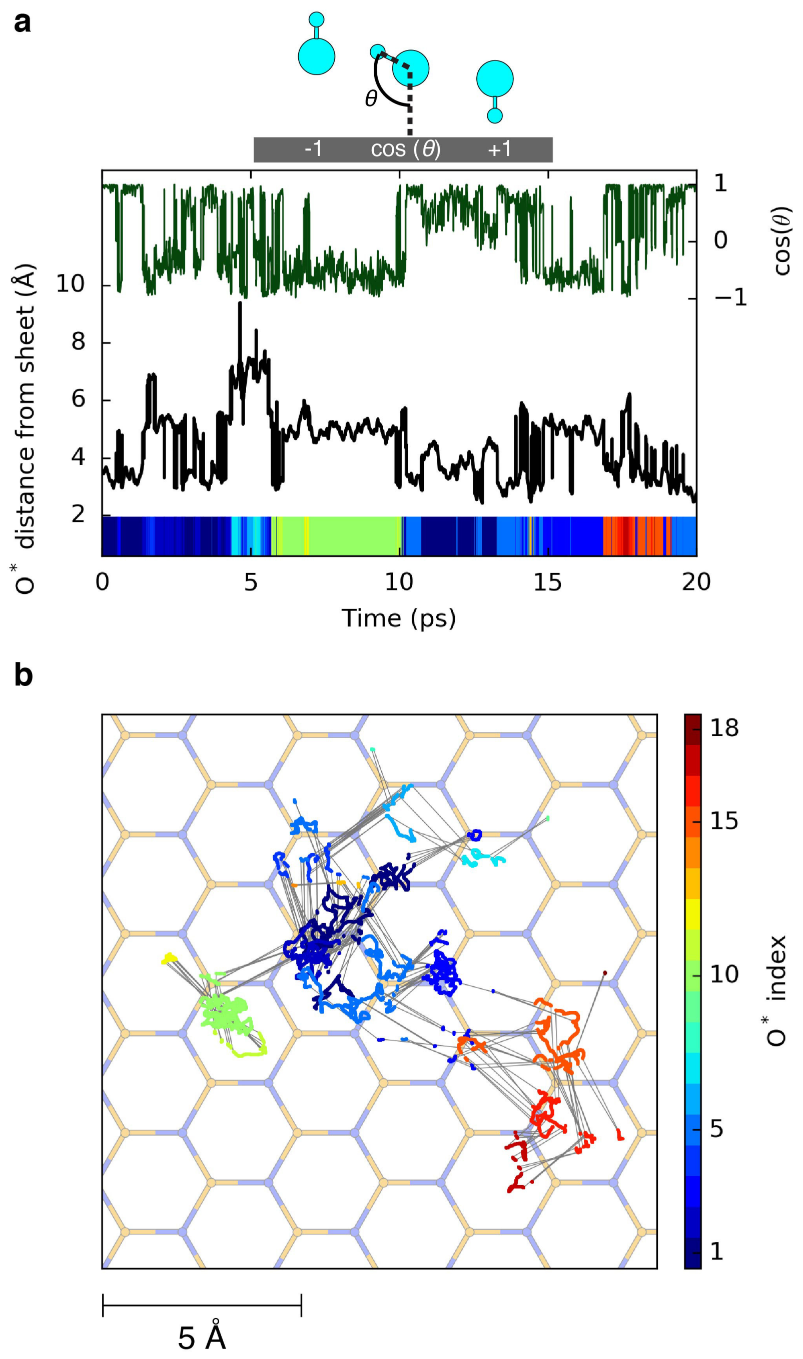

Supplementary Figure 5. Time variations of the vertical displacement (a, black curve) and lateral displacement (b, coloured trajectories) of a free hydroxide starting at a physisorption distance from a h-BN sheet. The cosine of the hydroxide orientation with respect to the surface is also plotted versus time (a, dark green curve). The index of the hydroxide oxygen O^* is associated to a specific colour so that changes of colour represent successive proton transfers in time and hence hydroxide diffusion. Proton transfers are also indicated by thin grey straight lines on top of the h-BN hexagonal grid represented in orange and blue (b).

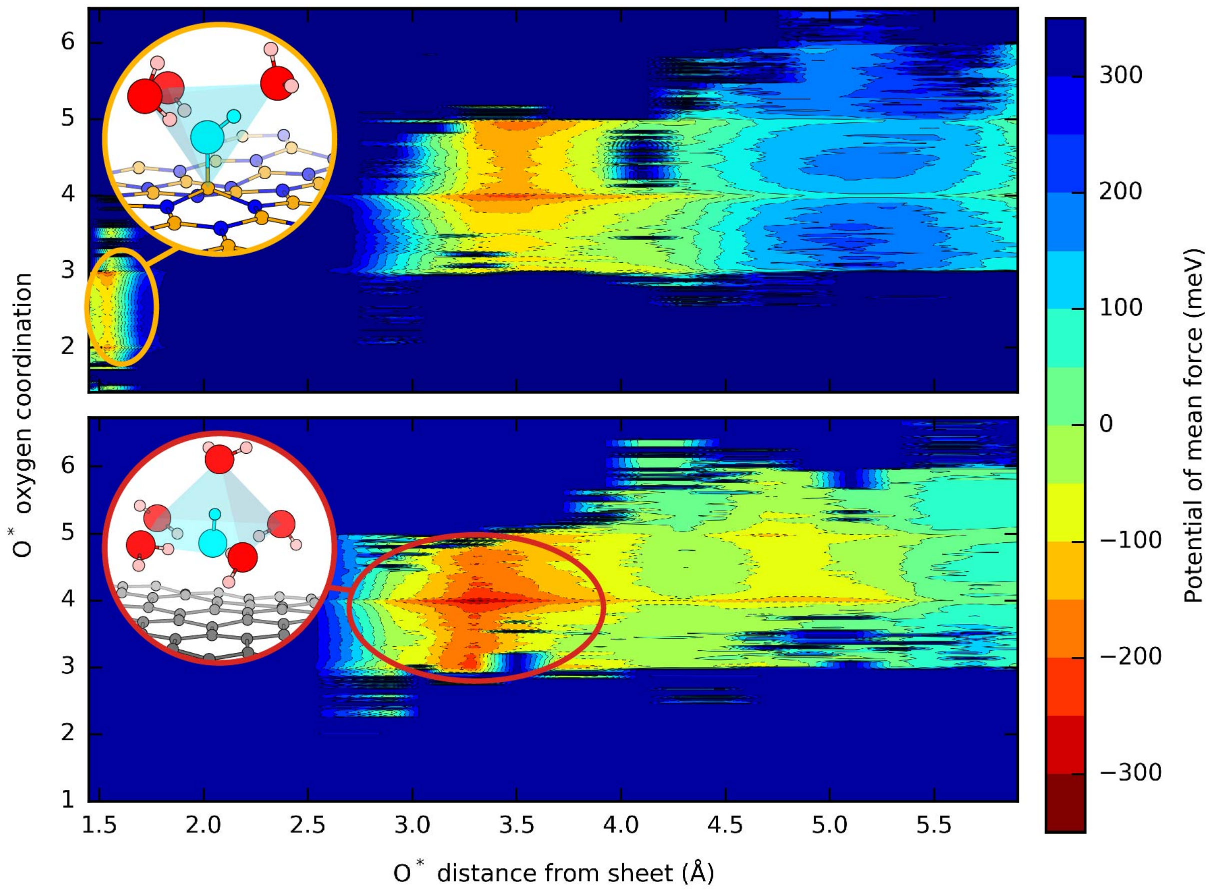

Supplementary Figure 6. Free energy profiles of the anion with respect to its oxygen coordination and its distance from a single layer h-BN (top) and graphene (bottom). The solvation configurations in insets correspond to the regions identified by ellipses: chemisorption on BN (top) and physisorption on graphene (bottom), with the hydroxide, hydrogen, boron, carbon, nitrogen and oxygen atoms respectively represented in cyan, pink, orange, gray, blue and red.

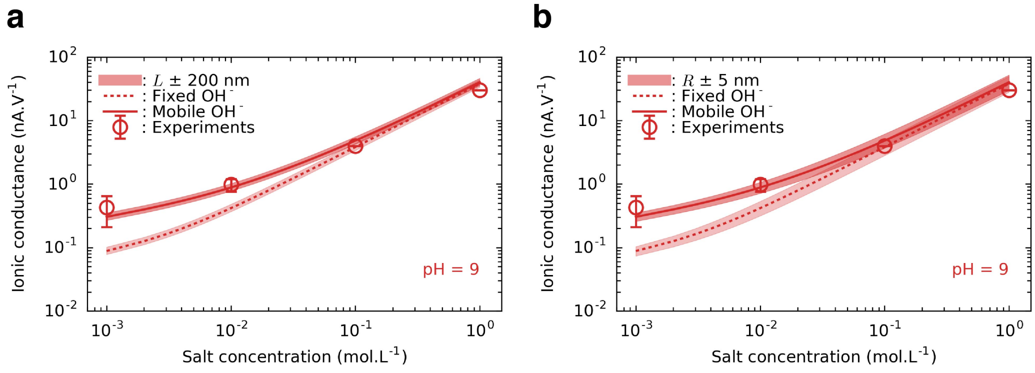

Supplementary Figure 7. Ionic conductance of a single 1500 nm long multiwall carbon nanotube with an inner diameter of 35 nm at pH 9 in function of salt concentration. Experimental measurements are represented by circles (courtesy of Secchi *et al.* [28]). The dotted and plain curves correspond to analytically derived conductance respectively including and excluding the hydroxide mobility. Colored areas correspond to the effect on the analytic models of the variations of 200 nm of tube length (a) and 5 nm of tube radius (b). Lower (upper) bound corresponds respectively to decreasing (increasing) the radius by 5 nm and to increasing (decreasing) the length by 200 nm.

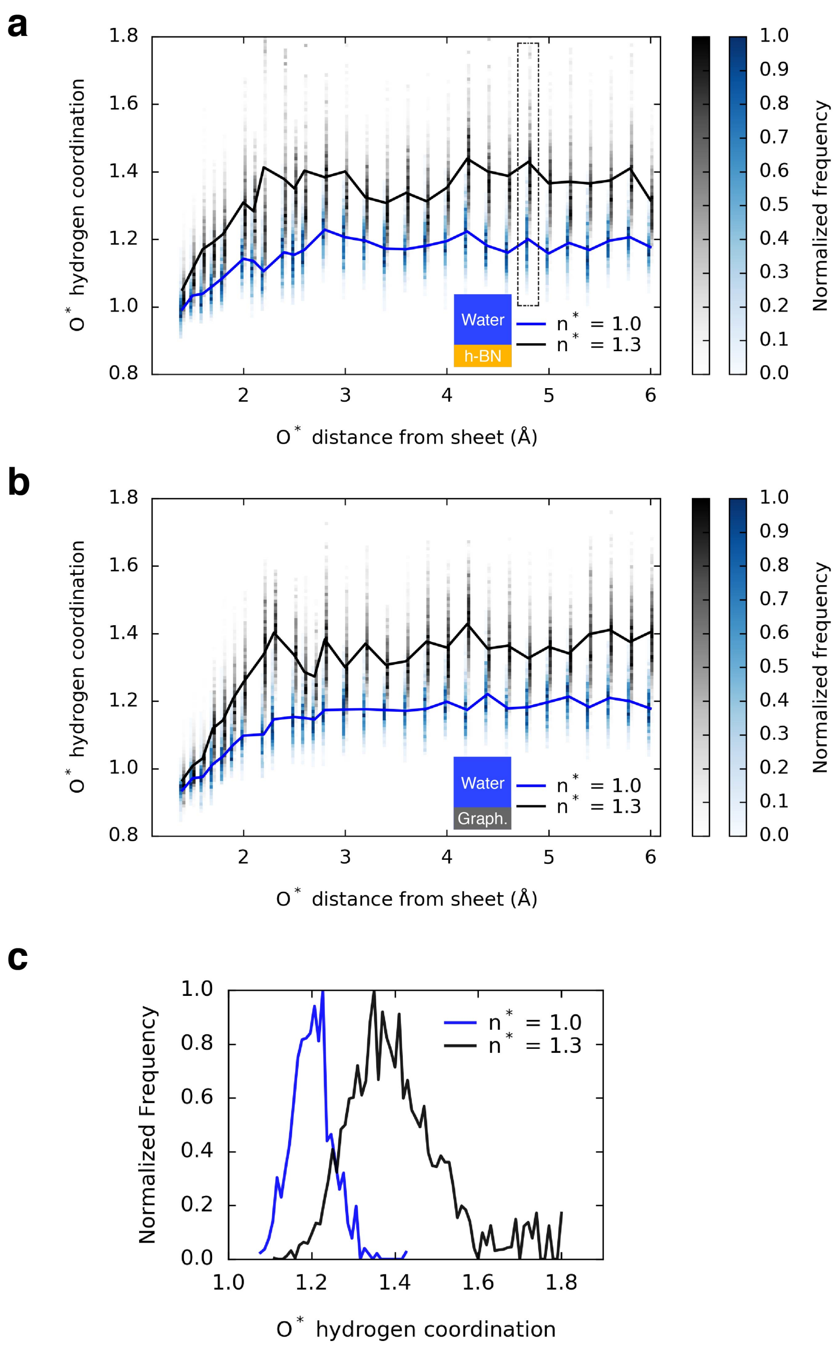

Supplementary Figure 8. Mean hydrogen coordination of OH^- at the water/h-BN (a) and water/graphene (b) interfaces for the two sets of MD trajectories respectively corresponding to a coordination restraint target n^* of 1.0 (blue) and 1.3 (black). Coordination histograms for each anion-surface distance are represented by vertical lines which color indicate the frequency of a particular coordination value following the color scales. (c) Overlap of two histograms obtained at a distance OH^--BN of 4.8 Å as identified by a dotted frame (a). Each histogram was normalized so that its maximum would be 1.0.

SUPPLEMENTARY REFERENCES

[1] CP2K Open Source Molecular Dynamics, https://www.cp2k.org/ (2018).

[2] VandeVondele, J. & Hutter, J. An efficient orbital transformation method for electronic structure calculations. *J. Chem. Phys.* **118**, 4365–4369 (2003).

[3] Vandevondele, J. *et al.* Quickstep: Fast and accurate density functional calculations using a mixed Gaussian and plane waves approach. *Comput. Phys. Commun.* **167**, 103–128 (2005).

[4] Frigo, M. & Johnson, S. The design and implementation of FFTW3. *Proc. IEEE* **93**, 216–231 (2005).

[5] Borštnik, U., Vandevondele, J., Weber, V. & Hutter, J. Sparse matrix multiplication: The distributed block-compressed sparse row library. *Parallel Comput.* **40**, 47–58 (2014). arXiv:1011.1669v3.

[6] Hutter, J., Iannuzzi, M., Schiffmann, F. & VandeVondele, J. cp2k: atomistic simulations of condensed matter systems. *Wiley Interdiscip. Rev. Comput. Mol. Sci.* **4**, 15–25 (2013).

[7] Lippert, G., Hutter, J. & Parrinello, M. A hybrid gaussian and plane wave density functional scheme. *Mol. Phys.* **92**, 477–488 (1997).

[8] VandeVondele, J. & Hutter, J. Gaussian basis sets for accurate calculations on molecular systems in gas and condensed phases. *J. Chem. Phys.* **127**, 114105 (2007).

[9] Goedecker, S., Teter, M. & Hutter, J. Separable Dual-Space Gaussian Pseudopotentials. *Phys. Rev. B* **54**, 1703–1710 (1996). 9512004v1.

[10] Hartwigsen, C., Goedecker, S. & Hutter, J. Relativistic separable dual-space Gaussian pseudopotentials from H to Rn. *Phys. Rev. B* **58**, 3641–3662 (1998).

[11] Krack, M. Pseudopotentials for h to kr optimized for gradient-corrected exchange-correlation functionals. *Theor. Chem. Acc.* **114**, 145–152 (2005).

[12] Perdew, J. P., Burke, K. & Ernzerhof, M. Generalized Gradient Approximation Made Simple. *Phys. Rev. Lett.* **77**, 3865–3868 (1996). 0927-0256(96)00008.

[13] Grimme, S., Antony, J., Ehrlich, S. & Krieg, H. A consistent and accurate ab initio parametrization of density functional dispersion correction (DFT-d) for the 94 elements h-pu. *J. Chem. Phys.* **132**, 154104 (2010).

[14] Grimme, S., Ehrlich, S. & Goerigk, L. Effect of the damping function in dispersion corrected density functional theory. *J. Comput. Chem.* **32**, 1456–1465 (2011).

[15] Nosé, S. A molecular dynamics method for simulations in the canonical ensemble. *Mol. Phys.* **52**, 255–268 (1984).

[16] Nosé, S. A unified formulation of the constant temperature molecular dynamics methods. *J. Chem. Phys.* **81**, 511–519 (1984).

[17] Visual Molecular Dynamics, http://www.ks.uiuc.edu/research/vmd/.

[18] Humphrey, W., Dalke, A. & Schulten, K. VMD – Visual Molecular Dynamics. *J. Mol. Graph.* **14**, 33–38 (1996).

[19] Stone, J. An efficient library for parallel ray tracing and animation. *Master Thesis, Comput. Sci. Dep., Univ. of Missouri-Rolla* (1998).

[20] Kumar, S., Rosenberg, J. M., Bouzida, D., Swendsen, R. H. & Kollman, P. A. The weighted histogram analysis method for free-energy calculations on biomolecules. *J. Comput. Chem.* **13**, 1011–1021 (1992).

[21] Souaille, M. & Roux, B. Extension to the weighted histogram analysis method: combining umbrella sampling with free energy calculations. *Comput. Phys. Commun.* **135**, 40–57 (2001).

[22] Allen, M. P. & Tildesley, D. J. *Computer Simulation of Liquids* (Oxford University Press, 2017).

[23] Lin, I.-C., Seitsonen, A. P., Tavernelli, I. & Rothlisberger, U. Structure and dynamics of liquid water from ab initio molecular dynamics—comparison of BLYP, PBE, and revPBE density functionals with and without van der waals corrections. *J. Chem. Theory Comput.* **8**, 3902–3910 (2012).

[24] Bankura, A., Karmakar, A., Carnevale, V., Chandra, A. & Klein, M. L. Structure, dynamics, and spectral diffusion of water from first-principles molecular dynamics. *J. Phys. Chem. C* **118**, 29401–29411 (2014).

[25] Bonthuis, D. J., Gekle, S. & Netz, R. R. Dielectric profile of interfacial water and its effect on double-layer capacitance. *Phys. Rev. Lett.* **107**, 166102 (2011).

[26] Horinek, D. & Netz, R. R. Specific ion adsorption at hydrophobic solid surfaces. *Phys. Rev. Lett.* **99**, 226104 (2007).

[27] Siria, A. *et al.* Giant osmotic energy conversion measured in a single transmembrane boron nitride nanotube. *Nature* **494**, 455–458 (2013).

[28] Secchi, E., Niguès, A., Jubin, L., Siria, A. & Bocquet, L. Scaling behavior for ionic transport and its fluctuations in individual carbon nanotubes. *Phys. Rev. Lett.* **116**, 154501 (2016).

SUPPLEMENTARY NOTES

Supplementary Note 1

Typical CP2K input file used for biased MD trajectories.

```
# CP2K input file example for Supplementary Information to: "Versatile Electrification
# of Two-dimensional Nanomaterials in Water"
@SET SYSTEM    system
@SET DATA_PATH /home/cp2k-data/
&FORCE_EVAL
  METHOD Quickstep
  &DFT
    CHARGE -1
    BASIS_SET_FILE_NAME ${DATA_PATH}/BASIS_SETS
    POTENTIAL_FILE_NAME ${DATA_PATH}/GTH_POTENTIALS
    &MGRID
      CUTOFF 600
    &END MGRID
    &QS
      EPS_DEFAULT 1.0E-14
      MAP_CONSISTENT TRUE
      EXTRAPOLATION ASPC
      EXTRAPOLATION_ORDER 4
    &END QS
    &PRINT
     &MULLIKEN
      FILENAME ${SYSTEM}
      &EACH
       MD 10
      &END EACH
     &END MULLIKEN
    &END PRINT
```

```
&SCF
  MAX_SCF 10
  SCF_GUESS RESTART
  EPS_SCF 1.0E-7
  &OUTER_SCF
    EPS_SCF 1.0E-7
    MAX_SCF 1000
  &END OUTER_SCF
  &OT ON
    MINIMIZER DIIS
    N_DIIS 5
  &END OT
&END SCF
&XC
  &XC_FUNCTIONAL PBE
  &END XC_FUNCTIONAL
  &vdW_POTENTIAL
     DISPERSION_FUNCTIONAL PAIR_POTENTIAL
     &PAIR_POTENTIAL
        TYPE DFTD3
        CALCULATE_C9_TERM .TRUE.
        REFERENCE_C9_TERM .TRUE.
        LONG_RANGE_CORRECTION .TRUE.
        PARAMETER_FILE_NAME ${DATA_PATH}/dftd3.dat
        VERBOSE_OUTPUT .TRUE.
        REFERENCE_FUNCTIONAL PBE
        R_CUTOFF [angstrom] 11.0
        EPS_CN 1.0E-6
     &END PAIR_POTENTIAL
  &END vdW_POTENTIAL
  &XC_GRID
    XC_SMOOTH_RHO NN50
```

```
      XC_DERIV NN50_SMOOTH
    &END
  &END XC
&END DFT
&SUBSYS
  &CELL
    ABC [angstrom] 12.83 12.35 21.0
  &END CELL
  &TOPOLOGY
    COORD_FILE_FORMAT xyz
    COORD_FILE_NAME ${SYSTEM}.xyz
  &END TOPOLOGY
    &COLVAR
      &COORDINATION
       ATOMS_FROM 122
       KINDS_TO H
 R0 [angstrom] 1.2
 NN 12
 ND 20
      &END COORDINATION
    &END COLVAR
  &KIND H
    BASIS_SET DZVP-MOLOPT-SR-GTH
    POTENTIAL GTH-PBE-q1
    MASS 2
  &END KIND
  &KIND O
    BASIS_SET DZVP-MOLOPT-SR-GTH
    POTENTIAL GTH-PBE-q6
  &END KIND
  &KIND B
    BASIS_SET DZVP-MOLOPT-SR-GTH
```

```
      POTENTIAL GTH-PBE-q3
    &END KIND
    &KIND N
      BASIS_SET DZVP-MOLOPT-SR-GTH
      POTENTIAL GTH-PBE-q5
    &END KIND
    &KIND C
     BASIS_SET DZVP-MOLOPT-SR-GTH
     POTENTIAL GTH-PBE-q4
    &END KIND
  &END SUBSYS
&END FORCE_EVAL
&GLOBAL
  PROJECT ${SYSTEM}
  RUN_TYPE MD
  PRINT_LEVEL LOW
  WALLTIME  82800
&END GLOBAL
&MOTION
   &MD
     ENSEMBLE NVT
     STEPS 10000
     TIMESTEP 0.5
     TEMPERATURE 323.15
     &THERMOSTAT
       &NOSE
         LENGTH 4
         YOSHIDA 9
         TIMECON [fs] 500.0
         MULTIPLE_TIME_STEPS 2
       &END NOSE
     &END THERMOSTAT
```

```
 &END MD
 &CONSTRAINT
   &FIXED_ATOMS
     COMPONENTS_TO_FIX XYZ
     LIST 122 43
   &END FIXED_ATOMS
  &COLLECTIVE
   COLVAR 1
   TARGET 1
   INTERMOLECULAR .TRUE.
   &RESTRAINT
    K 0.05
   &END RESTRAINT
  &END COLLECTIVE
 &END CONSTRAINT
&PRINT
  &TRAJECTORY  SILENT
    FILENAME =${SYSTEM}-1.xyz
    &EACH
      MD 1
    &END EACH
  &END TRAJECTORY
  &VELOCITIES  SILENT
    FILENAME =${SYSTEM}-1.vel
    &EACH
      MD 1
    &END EACH
  &END VELOCITIES
  &FORCES  SILENT
    FILENAME =${SYSTEM}-1.force
    &EACH
      MD 1
```

```
      &END EACH
    &END FORCES
    &RESTART
      FILENAME =${SYSTEM}-1.restart
      &EACH
        MD 1
      &END EACH
    &END RESTART
  &END PRINT
&END MOTION
```
