# Supplementary material for: Versatile electrification of two-dimensional nanomaterials in water
Source: Nat Commun. 2019 Apr 10;10:1656. doi: 10.1038/s41467-019-09708-7 (PMC6458114; doi:10.1038/s41467-019-09708-7)
Supplement: Supplementary file 3 — Description of Additional Supplementary Files [file 41467_2019_9708_MOESM3_ESM.pdf]

Description of Additional Supplementary Files

File Name: Supplementary Movie 1
Description: 20 ps long non-biased trajectory of a hydroxide physisorbed on h-BN in water. The hydroxide, hydrogen, boron, nitrogen and oxygen atoms are respectively represented in cyan, white, orange, blue and red.

File Name: Supplementary Movie 2
Description: 20 ps long non-biased trajectory of a hydroxide physisorbed on graphene in water. The hydroxide, hydrogen, carbon and oxygen atoms are respectively represented in cyan, white, grey and red.

File Name: Supplementary Movie 3
Description: 500 fs long non-biased trajectory of a hydroxide initially chemisorbed on h-BN (left) or graphene (right) in water. The hydroxide, hydrogen, boron, carbon, nitrogen and oxygen atoms are respectively represented in cyan, white, orange, grey, blue and red.
