# Supplementary material for: Versatile electrification of two-dimensional nanomaterials in water
Source: Nat Commun. 2019 Apr 10;10:1656. doi: 10.1038/s41467-019-09708-7 (PMC6458114; doi:10.1038/s41467-019-09708-7)
Supplement: Supplementary file 2 — Peer Review File [file 41467_2019_9708_MOESM2_ESM.docx]

Reviewers' comments:

Reviewer #1 (Remarks to the Author):

The authors apply state-of-the art ab initio simulation methods to calculate the interaction of a solvated OH ion to graphene and boron-nitride layers. The authors find that the ion adsorbs quite strongly at the BN and less strongly at the C surface, they also calculate the effective lateral conductivity and find strong enhancements due to a Grotthuis-like mechanism. The results are sound and interesting, in particular in relation to recent experimental measurements that found anomalous Zeta potentials in nanotubes. I therefore recommend publication of the paper and only have a few minor comments:

- adsorption potentials of ions at surfaces from the water phase are typically plotted with respect to the Gibbs dividing surface, it would be helpful to the reader if this dividing surface was included. Likewise, it would be helpful if the single-ion excess would be calculated and reported, i.e. the Botzmann weighted density with respect to the Gibbs dividing surface. Only when the surface excess is positive would one talk of ion adsorption !
- the underlying assumption is that the H3O ion adsorbs less strongly, this should be spelled out. At the air-water interface, the H3O ion has been shown to adsorb stronger than the OH ion, this should be discussed
- at many hydrophobic planar surfaces (I am not sure about the planar graphene surface) the Zeta potential is negative although from surface tension measurements it can be concluded that OH ions are repelled from hydrophobic surfaces. This seems at first sight to contradict the conclusions by the authors. This experimental finding has been recently quantitatively explained by adsorption of nano molar amounts of negatively charged surface-active impurities, see "Impurity effects at hydrophobic surfaces" Yuki Uematsu et al, Current Opinion in Electrochemistry (2018), https://doi.org/10.1016/j.coelec.2018.09.003. This might be interesting in the current context.
- there is a typo on line 78: fonction -> function Roland Netz

Reviewer #2 (Remarks to the Author):

I have read the article "Versatile Electrification of Two-dimensional Nanomaterials in Water" with great interest. This is a fascinating paper using detailed molecular level information to understand the mechanisms of ion transport in nano-channels. What I like is the simulation and the attention to detail with the protocol. The construction of the transport model pales in comparison (regarding detail) to the simulation data and that is really the central result of the paper. I would argue to put some of the mundane (but important) simulation data regarding the coordinate, etc to the SI. As written now, it is distracting and opens up the authors to a myriad of (important) technical and structural questions from the simulation community. This is distracting. I don't think the authors want this, and would rather focus on the science of the new phenomenon regarding transport of ions and the experiments.

As written now it is not suitable for the Nature Communications audience. I think this paper can run the distance. But it will need to be reorganized and refocused on the measurement and what molecular simulations bring to bear on reinterpreting the results.

Again, this is a great paper. t It could be a very influential paper and influence the way the community thinks about nanofluidics. These careful simulations warrant at least a couple of publications. There is a complete paper on the analysis of the structure, etc of the OH- at these

different surfaces. Nevertheless, the important result to be communicated is to discuss in detail the model for the transport and how molecular simulation helps shed light on how to use molecular level detail to augment the theory of transport.

I don't think this is a tall order for this excellent team. I think that the refocusing will make this a strong and influential paper.

Reviewer #3 (Remarks to the Author):

In this paper the authors studied the interaction hydroxide ions with boron nitride and graphene in contact with liquid water using ab initio MD

coupled with enhanced sampling techniques. This study greatly transcends their previous static calculations using implicit water only (JPCL 2016, 7, 4695).

That allows them do draw important conclusions as to experiments reporting puzzling and, so far, not understood results.

The authors study the interaction of a single hydroxide ion in explicit

water with a well chosen DFT method (PBE-D3, please replace PBE-DFTD3 by that standard nomenclature), which has been shown very recently to work well for water-graphene interactions (see comparison between PBE-D3 with DMC and CC studies in arXiv:1811.07645, which the authors should cite).

Very advanced statistical analyses of constrained MD trajectories were performed to extract the quantities of interest. The key conclusion is to show that enhanced ionic conductance at graphene sheets, so far a puzzling experimental finding without molecular explanation, can be

explained by physisorbed hydroxide ions which undergo 2D Grotthuss-like diffusion. This finding is novel and will, for sure, invoke a lot of interest.

Here is my list of suggestions, questions, and concerns in arbitrary order:

(1) the authors must comment on the pressure in their slab setup. It is known that water properties do depend on pressure, and the slab setup can easily shift the system quite a bit away from ambient (experimental) conditions with potential impact on water defect diffusion.
(2) Figure 1b is unclear to me: what is the meaning of the

two dotted lines since n* has been constrained to 1.0 or 1.3 and is, therefore, trivially constant, thus which observables are provided by the "two sets of MD trajectories (red dotted lines)

where computed with ...". Moreover, is the WHAM curve exclusively based on these two MD simulations (as a function of distance

to the surface)? If that is the case, the authors should provide evidence that the resulting histogram is sufficiently converged to provide the black curve given that (according to Fig. S2) a free OH- in bulk water (which certainly is approximately reached at a distance of 6 A from the surface) is subject to coordination number fluctuations up to about 1.8 (black curve in Fig. S2). In short, provide statistical evidence that the WHAM trajectories generated to obtain the PMFs in Fig. 2a are converged.

(3) the authors discuss on p. 9 the connection of the orientation of the OH- at the water/solid interface and its Grotthuss diffusion, which is crucial to explain the highly anisotropic ionic conductivity

at the surface. There is a study from the Marx group where this connection has already been analyzed in great detail in a bilayer

setup of another inert, hydrophobic surface (Nat Commun 2016, 7, 12625), whereas the current discussion is largely verbal and qualitative.

The authors should make an effort to analyze more quantitatively their free AIMD simulations in that respect, maybe including pre-solvation ideas in their specific case to understand Grotthuss migration as exposed by Tuckerman and coworkers in Chem Rev 2010, 110, 2174.

(4) the experimental motivation, reference [2], comes from measurements

within cylindrical CNTs whereas the authors study planar interfaces,

i.e. no confinement and no curvature. Nevertheless, the authors directly compare their computed conductance to the measured one. This needs quite a bit of explanation to support the idea of being able to compare the

very different geometries.

(5) also in relation to that experiment, the vastly different pH situations compared to the simulations must be addressed, in particular since the relative impact of pH on ionic conductance according to Fig. 5 is larger than the impact of mobile versus fixed OH- ions! I would also like to see something like a "stability analysis" of the prediction that culminates into the computed curves in that figure upon varying the parameters

in the model (in equations 3 and 4) that are needed to connect the PMF to the conductance. Moreover, it is unclear to me how the Grotthuss aspect of diffusion is folded into the modeling of OH- subject to

large surface mobility.

(6) a well-established concept in electrified aqueous interfaces is double layer formation. Assuming that a substantial number

of OH- ions get accumulated at the interface and, thus, are migrating essentially parallel to the graphene surface, it is expected that

the positive counterions might populate preferentially the subsequent solvation layer parallel to the surface. It is clear to me that

this effect cannot be included in the simulations for practical reasons, but based on which evidences and arguments can that approximation be justified?

(7) in the outlook, the authors speculate about interesting slit-pore nanofluid devices where only up to two water layers can penetrate. This is exactly what has already been studied for hydroxide (see again

Nat Commun 2016, 7, 12625), which suggests that the authors should combine these published insights with their novel findings to suggest experiments which could clarify that situation.

Again, I consider this manuscript to be potentially highly valuable, but I have to see signficant feedback of the authors on my points before I can come to a final recommendation.

*-	Reviewer #1 (Remarks to the Author):*

*The authors apply state-of-the art ab initio simulation methods to calculate the interaction of a solvated OH ion to graphene and boron-nitride layers. The authors find that the ion adsorbs quite strongly at the BN and less strongly at the C surface, they also calculate the effective lateral conductivity and find strong enhancements due to a Grotthus-like mechanism. The results are sound and interesting, in particular in relation to recent experimental measurements that found anomalous Zeta potentials in nanotubes. I therefore recommend publication of the paper and only have a few minor comments:*

We thank the reviewer for his positive review on our paper.

*- adsorption potentials of ions at surfaces from the water phase are typically plotted with respect to the Gibbs dividing surface, it would be helpful to the reader if this dividing surface was included. Likewise, it would be helpful if the single-ion excess would be calculated and reported, i.e. the Boltzmann weighted density with respect to the Gibbs dividing surface. Only when the surface excess is positive would one talk of ion adsorption !*

We thank the referee for proposing these additional concepts well-used in the domain of ionic adsorption at interfaces to complement our ab-initio derived PMF results.

Using a formula adapted from Ref. 28 (Bonthuis, PRL 2011, added to the main text) the Gibbs dividing surface has been evaluated to be positioned at 1.8 Å away from both hBN and graphene walls. And its trace for each material is now displayed in Fig. 2.a, as a blue vertical dotted line and the figure caption 2 was added the following sentence:

“The position of the Gibbs dividing surface(GDS), derived by adapting the calculation of Ref. 28 ,and the position of the free energy reference are respectively indicated by vertical blue and horizontal black dotted lines.”

Using the Boltzmann weighted density provided by the PMF, the single-ion excesses defined in Ref. 26 of the SI (Horinek, PRL 2007) were evaluated as positive values and precisely +64 µmol/m2 (+399 µmol/m^2^) for hBN (graphene). This corresponds to surface charges of respectively 6.2 C/m2 and 38.5 C/m2 higher than experimental values of Refs. 1 and 2 since the derivation of surface excesses is done in the limit of infinite dilution with no counter-ions, contrary to the model for ionic conductance. The following sentence was added to the main text (lines 107-109):

“While both free energy profiles yield positive surface excesses of hydroxides (see Supp. Info. Section II), there are striking differences between the two interfaces.”

We have added dedicated paragraphs regarding the Gibbs dividing surface and surface excesses in Supplementary Information, respectively section II.5. & II.6,.

- *the underlying assumption is that the H3O ion adsorbs less strongly, this should be spelled out. At the air-water interface, the H3O ion has been shown to adsorb stronger than the OH ion, this should be discussed*

We fully agree with the referee that we have to stress out that in our study we implicitly assume that the other self-ion of water namely the hydronium ion adsorbs less strongly than the hydroxide. We indeed make use of the measurements regarding variations of pH between 6 and 9 (taken from Ref. 2 and shown in Fig. 5) that remain a

strong advocate for a preferential adsorption of hydroxides at the interface with respect of hydronium counterion.

We are planning to conduct a related adsorption study of hydronium on those materials as well. We have added a short sentence in the introduction to stress that argument line 68 :

- Herein we report AIMD-based calculations of the free energy of a hydroxide at the water-graphene and the water-BN interfaces, “considering that the clear experimental pH dependency of the surface charge infers a charging due to the anionic self-ion of water rather than its hydronium counterpart.**”**

- *at many hydrophobic planar surfaces (I am not sure about the planar graphene surface) the Zeta potential is negative although from surface tension measurements it can be concluded that OH ions are repelled from hydrophobic surfaces. This seems at first sight to contradict the conclusions by the authors. This experimental finding has been recently quantitatively explained by adsorption of nanomolar amounts of negatively charged surface-active impurities, see "Impurity effects at hydrophobic surfaces" Yuki Uematsu et al, Current Opinion in Electrochemistry (2018), https://doi.org/10.1016/j.coelec.2018.09.003. This might be interesting in the current context.*

We thank the referee for pointing this novel work allowing reconciling experiments and state-of-the-art modelling of the air-water interface by considering additional adsorption of trace amount anionic impurities in the water media. In our membrane-water interface we don’t see any conflicting picture since not only the sign of the Zeta potential but also the strong dependence of the charge magnitude on pH corroborate a charging mechanism based on the adsorption of the majority anionic species namely the hydroxide.

However we make a short mention in the main draft and add the reference :

page 3 line 66 : ...the water-air interface [18,22,23]. “Also, for this interface, comparison with experiments is delicate because of impurity effects[24] [Ref :24]**”**

- *there is a typo on line 78: fonction -> function*

It is corrected.

Roland Netz

*-	Reviewer #2 (Remarks to the Author):*

*I have read the article "Versatile Electrification of Two-dimensional Nanomaterials in Water" with great interest. This is a fascinating paper using detailed molecular level information to understand the mechanisms of ion transport in nano-channels. What I like is the simulation and the attention to detail with the protocol. The construction of the transport model pales in comparison (regarding detail) to the simulation data and that is really the central result of the paper. I would argue to put some of the mundane (but important) simulation data regarding the coordinate, etc to the SI. As written now, it is distracting and opens up the authors to a myriad of (important) technical and structural questions from the simulation community. This is distracting. I don't think the authors want this, and would rather focus on the science of the new phenomenon regarding transport of ions and the experiments.*

We thank the referee for his high-quality comments regarding our paper. And his advice proposing to place our complex simulation protocol in SI is crucial to emphasize at best our central results and we thank him for aiming at improving the paper’s impact. Followingly, technical details regarding simulations were moved to SI (sections I.1) and more attention and extended details on the transport phenomenon model have been provided in section entitled “Comparison to conductance…” line 192, with emphasis of the model for the surface charge contribution to the overall conductance. We detail the additions to this section in our answers to referee#3 (see below answers to point 4 & 5).

*As written now it is not suitable for the Nature Communications audience. I think this paper can run the distance. But it will need to be reorganized and refocused on the measurement and what molecular simulations bring to bear on reinterpreting the results.*

*Again, this is a great paper. t It could be a very influential paper and influence the way the community thinks about nanofluidics. These careful simulations warrant at least a couple of publications. There is a complete paper on the analysis of the structure, etc of the OH- at these different surfaces. Nevertheless, the important result to be communicated is to discuss in detail the model for the transport and how molecular simulation helps shed light on how to use molecular level detail to augment the theory of transport.*

*I don't think this is a tall order for this excellent team. I think that the refocusing will make this a strong and influential paper.*

Thanks again for the compliments and we hope that the focusing we have made in the revised draft will comply with the referee propositions.

*-Reviewer #3 (Remarks to the Author):*

*In this paper the authors studied the interaction hydroxide ions with boron nitride and graphene in contact with liquid water using ab initio MD coupled with enhanced sampling techniques. This study greatly transcends their previous static calculations using implicit water only (JPCL 2016, 7, 4695). That allows them do draw important conclusions as to experiments reporting puzzling and, so far, not understood results.*

*The authors study the interaction of a single hydroxide ion in explicit water with a well chosen DFT method (PBE-D3, please replace PBE-DFTD3 by that standard nomenclature), which has been shown very recently to work well for water-graphene interactions (see comparison between PBE-D3 with DMC and CC studies in arXiv:1811.07645, which the authors should cite). Very advanced statistical analyses of constrained MD trajectories were performed to extract the quantities of interest. The key conclusion is to show that enhanced ionic conductance at graphene sheets, so far a puzzling experimental finding without molecular explanation, can be explained by physisorbed hydroxide ions which undergo 2D Grotthuss-like diffusion. This finding is novel and will, for sure, invoke a lot of interest.*

We thank the referee for pointing out the novelty of our findings.

The reference (Brandenburg *et al.* 2018) was added to the Methods section :

“PBE-D3 has indeed been recently shown to provide a good description of the water/graphene interface[47].”

We have done the replacements of DFT acronyms in order to comply with the nomenclature page 9 Table 1, in the Methods section and in SI section I.

*Here is my list of suggestions, questions, and concerns in arbitrary order:*

(1) *the authors must comment on the pressure in their slab setup. It is known that water properties do depend on pressure, and the slab setup can easily shift the system quite a bit away from ambient (experimental) conditions with potential impact on water defect diffusion.*

The referee is right that the computed pressure needs to be reported in each simulation cell and have computed it now. To do so the pressure has been evaluated on the 20 ps free trajectories using the following protocol that we also detail in SI section I.2.a: the forces were recomputed on 200 snapshots distant of 0.1 ps and in which a 10 Å vacuum separation was added between water and one side of the atomic layer. The pressure was then

determined as the average over the 200 snapshots of the sum of normal forces on surface atoms divided by the simulation cell section area.

Followingly we obtain a mean pressure of 0 ± 93 MPa in the simulation cell with hBN and 73 ± 95 MPa, the latter value being on the high side but including the desired pressure within its error bars. We also checked the radial distribution functions of water in bulk water or confined between atomic sheets and found no observable variations of the overall liquid structure.

(2) *Figure 1b is unclear to me: what is the meaning of the two dotted lines since n* has been constrained to 1.0 or 1.3 and is, therefore, trivially constant, thus which observables are provided by the "two sets of MD trajectories (red dotted lines) where computed with ...". Moreover, is the WHAM curve exclusively based on these two MD simulations (as a function of distance to the surface)? If that is the case, the authors should provide evidence that the resulting histogram is sufficiently converged to provide the black curve given that (according to Fig. S2) a free OH- in bulk water (which certainly is approximately reached at a distance of 6 A from the surface) is subject to coordination number fluctuations up to about 1.8 (black curve in Fig. S2). In short, provide statistical evidence that the WHAM trajectories generated to obtain the PMFs in Fig. 2a are converged.*

Figure 1b was indeed not clear-cut and has now been strongly modified. In general PMFs are built by solely constraining the ion-material distance (horizontal axis). Here because of the transient nature of the hydroxide one needs to add a restraint on its coordination (vertical axis) to preserve it and this restraint is in our system spatially inhomogeneous, ranging from 1 near the material to 1.3 in the bulk water. To better illustrate this, the horizontal lines displaying the two target values for the restraint concerning the hydroxide coordination are now replaced by the mean value of the coordination and the associated standard deviation, for the two sets of restrained MD trajectories at each hydroxide-material distance.

In addition, as the referee questions it, the WHAM analysis is indeed based on the two restrained MD simulations. And we now provide statistical evidence of the convergence of the WHAM analysis in the SI (section II.1), with SI Fig. 5 displaying the mean coordination of the hydroxide and its probability distribution for each distance and for each restraint target value, to visualize the overlap between each couple of histograms on which the WHAM analysis is performed.

(3) *the authors discuss on p. 9 the connection of the orientation of the OH- at the water/solid interface and its Grotthuss diffusion, which is crucial to explain the highly anisotropic ionic conductivity at the surface. There is a study from the Marx group where this connection has already been analyzed in great detail in a bilayer setup of another inert, hydrophobic surface (Nat Commun 2016, 7, 12625), whereas the current discussion is largely verbal and qualitative. The authors should make an effort to analyze more quantitatively their free AIMD simulations in that respect, maybe including pre-solvation ideas in their specific case to understand Grotthuss migration as exposed by Tuckerman and coworkers in Chem Rev 2010, 110, 2174.*

The two references are indeed meaningful for our work and have been added to the main text. (Ref. 19 and Ref. 36). We agree that we did not analyze in such quantitative details our free AIMDs. Indeed we want to stress more on the further rationalization of the experiments by the newly found transport mechanism, which is - as noted by referee #2 - the main result of our work. However we will analyze better the molecular motion of OH- and other ions such as H3O+ - as suggested by referee #1 - in a forthcoming publication.

(4) *the experimental motivation, reference [2], comes from measurements within cylindrical CNTs whereas the authors study planar interfaces, i.e. no confinement and no curvature. Nevertheless, the authors directly compare their computed conductance to the measured one. This needs quite a bit of explanation to support the idea of being able to compare the very different geometries.*

Indeed, our simulation system do not include curvature as the experimental ones have considered a range of small curvatures (with tube radii varying from 3.5 to 35 nm in the works of Secchi et al. (Ref. 1 : PRL, 2016, see Fig. caption 1). In particular, a flat material is a good model geometry of the CNT radius of 35 nm whose conductivity measurements were compared to in Fig. 5. It is noteworthy that periodic images of the simulated planar surfaces are distant of 21 Å so that confinement is in fact taken into account.

We have added a short sentence in the text about the absence of curvature effect in our model : page 8 lines 199-202:

“Since the tubes's diameters range between 3.5 nm and 35 nm, the curvature effect remains subtle. Hence our simulated planar surfaces confining water in a space of around 2 nm width are appropriate atomistic models of the measured tubes.”

(5) *also in relation to that experiment, the vastly different pH situations compared to the simulations must be addressed, in particular since the relative impact of pH on ionic conductance according to Fig. 5 is larger than the impact of mobile versus fixed OH- ions! I would also like to see something like a "stability analysis" of the prediction that culminates into the computed curves in that figure upon varying the parameters in the model (in equations 3 and 4) that are needed to connect the PMF to the conductance. Moreover, it is unclear to me how the Grotthuss aspect of diffusion is folded into the modeling of OH- subject to large surface mobility.*

Figure 5 has been modified to include an additional plot and caption that illustrate the effect of a pH difference of 1 unit on the model including the extra mobility of OH-, displayed by colored area. For sake of clarity, the comparative effects of tube radius and length are displayed in a new figure in SI (Supplementary Figure 8).

The Grotthus mechanism is incorporated in our model mainly through the large mobility of the adsorbed hydroxide.

We have added an extra discussion in the text page 13 related to the new Figure 5 lines 246-253:

“We have checked the stability of the predicted conductance with respect to variations in the parameters of the models. The effects of variations in R or L are limited (see Supp. Info. section II), while it is the largest for variations in pH. The effect of a pH variation of one unit is displayed in Fig.5 right panel for the two models, including or not the surface charge mobility. The two predictions are still clearly separated showing that the effect of surface charge mobility is significant even with respect to variations of other parameters in the model. The effect of variations of the geometry of the tube are shown in Supp. Fig. 8.”

(6) *a well-established concept in electrified aqueous interfaces is double layer formation. Assuming that a substantial number of OH- ions get accumulated at the interface and, thus, are migrating essentially parallel to the graphene surface, it is expected that the positive counterions might populate preferentially the subsequent solvation layer parallel to the surface. It is clear to me that this effect cannot be included in the simulations for practical reasons, but based on which evidences and arguments can that approximation be justified?*

The referee is fully right that positive counterions have to compensate the negative surface charge unveiled by our study and these residual positive counterions are responsible for the residual conductance at zero salt concentrations.

The reason why we did not include counterions in our simulations is that we first questioned whether the hydroxide ion in plain water has an affinity or not on these materials and this independently of the presence of the other ions. But indeed we plan to investigate one step further i.e. mimicking the interface with realistic electrolytes containing salts.

However, while these effects are thus completely absent from our simulations, they are incorporated in an effective way in the charge equalization model used to compare with experiments. This is done through a continuous description of the charge density in the solution, using the Poisson-Boltzmann equation. We have incorporated a discussion of this point in the text page 11 lines 203-209 :

“In order to compare our results with experiments, it is necessary to take into account the electrostatic repulsion between adsorbed hydroxide ions and the presence of counterion in the double layer of the surface for

charge equalization. These effects are missing in our simulation of a single hydroxide ion in absence of any counterion. Such effects however have been modeled successfully using mean-field approaches[34]”.

(7) *in the outlook, the authors speculate about interesting slit-pore nanofluid devices where only up to two water layers can penetrate. This is exactly what has already been studied for hydroxide (see again Nat Commun 2016, 7, 12625), which suggests that the authors should combine these published insights with their novel findings to suggest experiments which could clarify that situation.*

We thank the referee for pointing out this simulation study using inorganic slit-pore devices. We have now added the reference in our conclusion. Similarly to this paper we are studying the confinement effect where water is only defined by one or two layers inserted between our organic materials, in line with the recent measurements performed in the group of A. Geim (*Nature* paper in press: “Molecular streaming and voltage-gated response in Angström scale channels” T. Mouterde, A. Keerthi, A. Poggioli, S. Dar, A. Siria, A.K. Geim, L Bocquet and R. Boya, Nature (2019) in press).

We will also tentatively compare our water behaviour confined between organic sheets to the one confined between inorganic sheets noted by the referee.

In the last sentence of our manuscript, we add:

“in the same spirit of the recent hydroxide solvation modelling in inorganic mackinawite slit-pores [Ref. 35].”

*Again, I consider this manuscript to be potentially highly valuable, but I have to see significant feedback of the authors on my points before I can come to a final recommendation.*

We hope that we have clarified all the questions raised by the referee and have showed that this work is entirely new.

With the revision of the main text and SI we feel that we now emphasize better the unforeseen concept of hydroxide mobility thanks to the combination of preferential surface charging on prototypical 2D materials and of the Grotthus mechanism within the first layer of water molecules.

REVIEWERS' COMMENTS:

Reviewer #1 (Remarks to the Author):

The authors have addressed all my comments, I happily recommend publication as is. Roland Netz

Reviewer #2 (Remarks to the Author):

The authors did a great job on answering concerns of all reviewers. Publish without further delay.

Reviewer #3 (Remarks to the Author):

I carefully worked through the changes made and the reply letter.

My conclusion is that the authors did answer all my questions

in a more than satisfactory way, modifying the MS and SI wherever necessary appropriately.

I also think that the MS improved much compared to its original version, thus making it much more easily accessible to the broad readership of Nat Commun. The SI is now much more comprehensive and provides the required support of the key results.

Therefore, I strongly recommend publication of this submission in its current version.

Dominik Marx
